# A Mesophilic Argonaute from *Cohnella algarum* Mediates Programmable DNA/RNA Cleavage with Distinctive Guide Specificity

**DOI:** 10.3390/biom15101459

**Published:** 2025-10-16

**Authors:** Yanhong Peng, Wang Pan, Yang Wang, Yang Liu, Lixin Ma

**Affiliations:** State Key Laboratory of Biocatalysis and Enzyme Engineering, Hubei Key Laboratory of Industrial Biotechnology, School of Life Sciences, Hubei University, Wuhan 430062, China; pyhong@stu.hubu.edu.cn (Y.P.); 202421107011831@stu.hubu.edu.cn (W.P.); lyang@hubu.edu.cn (Y.L.)

**Keywords:** mesophilic Argonaute, DNA and RNA targeting, distinctive guide specificity

## Abstract

Argonaute (Ago) proteins are ubiquitous across all domains of life. Some prokaryotic Agos (pAgos) function as endonucleases that utilize short nucleic acid guides to recognize and cleave complementary targets. Yet, considerable diversity within pAgos leaves many of their biochemical and functional features insufficiently understood. This study characterizes CalAgo, an pAgo from the mesophilic bacterium *Cohnella algarum*, which demonstrates DNA-guided DNA endonuclease and RNA endonuclease activities at physiological temperatures. CalAgo’s cleavage activity depends on Mn^2+^ and Mg^2+^ ions and remains effective across a wide range of temperatures and pH levels. CalAgo utilizes only short guides ranging from 15 to 21 nucleotides (nt) in length, in contrast to other reported pAgos that target both DNA and RNA, which often exhibit broad guide selectivity. CalAgo preferentially loads 5′-phosphorylated guides and shows no significant preference among guides with different 5′-end nucleotides. CalAgo is sensitive to guide–target mismatches, and introducing a single mismatch at positions 12 or 15 of the guide strand abolished detectable activity. Structural modeling suggests that this unique guide specificity may originate from structural features in its PAZ domain involved in 3′-guide binding. In summary, this study deepens insight into mesophilic pAgos and supports their potential utility in nucleic acid-based applications.

## 1. Introduction

Argonaute (Ago) proteins constitute a conserved class of nucleic acid-binding proteins that exhibit significant evolutionary and functional divergence between prokaryotes and eukaryotes [1]. Eukaryotic Ago proteins (eAgos) play central roles in antiviral defense and gene regulation [2]. Their canonical mechanism involves binding small non-coding RNAs (sncRNAs) to guide the binding/cleavage of complementary RNA targets, thereby facilitating gene silencing [3,4]. Prokaryotic Ago proteins (pAgos) are primarily disseminated through horizontal gene transfer [5,6] and function as defense systems in bacteria and archaea against foreign genetic elements [7,8].

Phylogenetic analyses classify pAgos into three major clades: long pAgos (subdivided into long-A and long-B), short pAgos, and PIWI-RE proteins [6,9,10]. Long pAgos exhibit structural similarity to their eukaryotic counterparts, comprising four conserved domains: the N-terminal (N), middle (MID), PIWI, and PAZ (PIWI–Ago–Zwille) domains [7]. In contrast, short pAgos are truncated variants that lack the N and PAZ domains [11]. These domains exhibit specialized functional division: the MID and PAZ domains anchor the 5′- and 3′-ends of the guide nucleic acid, respectively [12], while the PIWI domain, adopting an RNase H-like fold, utilizes a conserved DEDX (where X = D, H, N, or K) catalytic tetrad to coordinate divalent metal ions. This enables cleavage of the target nucleic acid between nucleotides 10 and 11 relative to the guide’s 5′-end [6,7,13,14]. The N domain, displaying the lowest sequence conservation, facilitates processes such as product release and guide duplex dissociation [14,15]. Structural studies reveal that in both eAgos and long pAgos, the N-PAZ and MID-PIWI lobes form a bilobal architecture, forming the binding interface for the guide–target nucleic acid complex [12].

pAgos demonstrate extensive catalytic diversity. Initial research focused predominantly on thermophilic pAgos [16,17,18,19], which utilize DNA guides (gDNAs) to cleave DNA or RNA targets but exhibit limited activity under moderate temperatures. Recent discoveries of mesophilic pAgos have significantly broadened their potential applications [20]. Notably, pAgos display considerable variation in substrate preference: while most employ DNA guides [10,21,22], *Marinitoga piezophila* Ago (MpAgo) [23] and Candidatus *Harpocratesius repetitus* Ago1 (HrAgo1) [24] utilize RNA guides; the majority cleave DNA targets, whereas enzymes like *Mucilaginibacter paludis* Ago (MbpAgo) [25] and *Pseudooceanicola lipolyticus* Ago (PliAgo) [26] preferentially cleave RNA. Furthermore, some pAgos, such as *Thermus thermophilus* Ago (TtAgo) [18] and *Kurthia massiliensis* Ago (KmAgo) [27,28], are capable of targeting both DNA and RNA. This functional versatility positions pAgos as promising tools for nucleic acid detection [29], molecular cloning [30], and genome editing [9,31]. Unlike the CRISPR-Cas9 system, which directly cleaves double-stranded DNA (dsDNA) [32], pAgos typically require DNA helicase assistance for dsDNA cleavage, facilitating independent nicking events on each strand [33]. Additionally, their intrinsic non-specific nuclease activity adds a further layer of regulatory complexity [19,21,34,35].

Despite the considerable promise of mesophilic pAgos in biotechnology, current research faces two major limitations: (1) significant discrepancies exist between phylogeny-based functional predictions and experimentally determined activities; and (2) the structure–function relationships of the N-terminal and PAZ domains remain poorly understood. This study presents a systematic biochemical characterization of CalAgo, a DNA- and RNA-targeting pAgo derived from *Cohnella algarum*. Key parameters investigated include its temperature/pH/ion requirements, guide length dependency, 5′-end nucleotide preference, and mismatch tolerance. These findings expand the mesophilic pAgo toolkit and provide a foundation for the rational design of next-generation Ago-based molecular tools.

## 2. Materials and Methods

### 2.1. Bacterial Strains and Growth Conditions

*Escherichia coli* DH5α was employed for standard plasmid construction, while *E. coli* BL21(DE3) served as the expression host. All strains were cultured in Luria–Bertani (LB) medium.

### 2.2. Sequence Analysis

To discover new potential pAgo candidates, BLAST v2.15.0, based on KmAgo amino acid sequences, was performed in the NCBI database, and the characterized long-A pAgo sequences were selected for phylogenetic analysis using MEGA 11.0 [36]. For the active site residue sequence alignment, Ago sequences from *Kurthia massiliensis* (KmAgo, WP_010289662.1), *Metasolibacillus* sp. FSL K6-0083 (MeAgo, WP_342558927.1), *Domibacillus enclensis* (DeAgo, WP_052698403.1), *Rummeliibacillus suwonensis* (RsuAgo, WP_146547607.1), *Bacillus* sp. FJAT-42315 (BsAgo, WP_100402176.1), *Intestinibacter bartlettii* (IbAgo, WP_055087491.1), *Exiguobacterium marinum* DSM-16307 (EmaAgo, WP_026824436.1), *Exiguobacterium* sp. AB2 (EsAgo, WP_034806158.1), *Paenibacillus borealis* (PbAgo, WP_042211195), and *Brevibacillus laterosporus* (BlAgo, WP_096885432) were aligned with the muscle. Residues near the DEDX catalytic tetrad were highlighted in yellow.

### 2.3. CalAgo Expression and Purification

The His-tagged (C-terminal) CalAgo (Accession ID: MBN2983234) gene with optimized codons was synthesized and cloned into the pET23a plasmid to yield the expression plasmid pET23a-CalAgo by Tsingke Biotech (Beijing, China). Catalytically mutant (CalAgo-DM, D502A/D571A) was introduced by site-directed mutagenesis using T5 exonuclease-mediated low-temperature DNA cloning method [37] and verified by DNA sequencing.

CalAgo and CalAgo-DM were expressed in *E. coli* BL21 (DE3) host strain and purified as previously described [27]. The expression of recombinant protein was induced by 1 mM isopropyl-beta-D-thiogalactopyranoside (IPTG) at 18 °C for 16 h, and the resulting protein was purified using an Ni-NTA affinity column and Heparin column. Specifically, cell pellets were resuspended in Buffer A [20 mM Tris–HCl (pH 7.5), 500 mM NaCl, 20 mM imidazole] containing 1 mM phenylmethylsulfonyl fluoride (PMSF), and lysed by high-pressure homogenization. After centrifugation, the supernatant was incubated with Ni–NTA Beads 6FF gravity column (Smart-Lifesciences, Changzhou, China) for 1.5 h at 4 °C under gentle rotation. Bound proteins were washed with Buffer A and eluted with Buffer A containing 50 mM and 150 mM imidazole. CalAgo-containing fractions were concentrated using an Amicon Ultra-50K filter (Millipore, Billerica, MA, USA) and diluted with 20 mM HEPES (pH 7.5) to adjust NaCl concentration to 125 mM. The diluted protein was loaded onto a HiTrap Heparin HP column (GE Healthcare, Chicago, IL, USA) equilibrated with Buffer C (20 mM HEPES, pH 7.5, 125 mM NaCl), washed, and eluted with a linear NaCl gradient (0.125–2 M). Purity was confirmed by SDS-PAGE. The purified protein was concentrated, buffer-exchanged into Buffer B, aliquoted, and flash-frozen in liquid nitrogen for storage.

Protein structure prediction was performed with AlphaFold3 algorithm using AlphaFold Server [38]. The best model for each protein was visualized with PyMol v3.1.6.1.

### 2.4. Enzymatic Activity Assay

Standard cleavage assays were typically conducted using a 6:2:1 molar ratio of CalAgo, guide, and target at 37 °C for 30 min, unless stated otherwise. For complex assembly, 1.2 µM CalAgo was preincubated with 0.4 µM guide strand in reaction buffer (10 mM HEPES–NaOH, pH 7.5, 100 mM NaCl, 0.5 mM MnCl_2_, and 5% glycerol) at 37 °C for 10 min to facilitate guide loading. The reaction was initiated by adding the target strand to a final concentration of 0.2 µM. At specified time points, reactions were quenched by mixing with an equal volume of 2× denaturing loading buffer (95% formamide, 18 mM EDTA, 0.025% SDS, and 0.025% bromophenol blue), followed by incubation at 95 °C for 5 min. Cleavage products were separated on 20% urea-PAGE gels. For quantitative analysis, FAM-labeled targets were employed, as this fluorophore provides improved accuracy in calculating cleavage efficiency compared with SYBR Gold staining. Fluorescent signals were detected using the GelDoc Go imaging system (Bio-Rad, Hercules, CA, USA) under SYBR Gold settings. Band intensities were quantified with ImageJ v1.53k (NIH, Bethesda, MD, USA), and data visualization was performed using GraphPad Prism v8.3.1 (GraphPad Software, La Jolla, CA, USA). Cleavage efficiency (%) was defined as the intensity of the product band divided by the sum of product and remaining substrate intensities.

To assess temperature dependence, reactions were incubated at different temperatures simultaneously in a PCR thermocycler (Bio-Rad, Hercules, CA, USA). For cation dependency tests, Mn^2+^ in the buffer was substituted with other divalent metal ions at equal concentrations. The effect of the 5′-terminal base of guide DNA was examined using gDNAs carrying 5′-C, 5′-T, 5′-A, or 5′-G nucleotides. The optimal gDNA length adopted by CalAgo was measured in the presence of 8–30 nt gDNA. A single mismatch was introduced in the gDNA from positions 1–18, respectively, to test the guide–target mismatch tolerance. All cleavage experiments were performed in triplicates. All nucleic acids used in this study are listed in Table S1.

### 2.5. Electrophoretic Mobility Shift Assay (EMSA)

Electrophoretic mobility shift assays (EMSA) were performed to assess the loading of guide–target duplexes by CalAgo_DM. Reaction mixtures (20 µL) contained 128 nM 5′OH-gDNA, 64 nM 5′-FAM-labeled target strand, varying concentrations of CalAgo_DM, 20 mM HEPES–NaOH (pH 7.5), 100 mM NaCl, and 10 mM MnCl_2_. Samples were incubated at 37 °C for 30 min to allow complex formation, followed by the addition of 2.2 µL of 10× loading buffer (250 mM Tris–HCl, pH 7.5, 40% glycerol). The reaction products were resolved on 10% native polyacrylamide gels prepared in 0.5× TBE buffer, and fluorescent signals were visualized using a Gel Doc Go imaging system (Bio-Rad).

## 3. Results

### 3.1. Sequence Analysis of CalAgo

**Figure 1:**
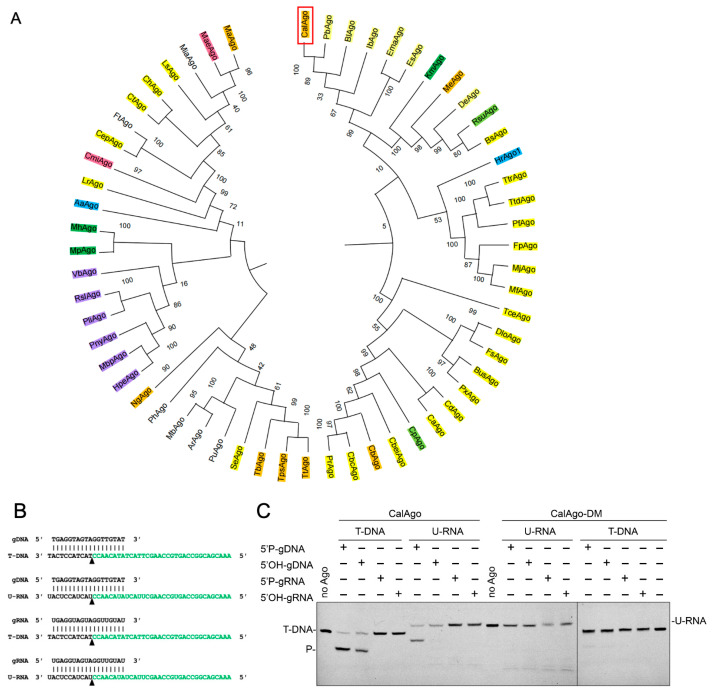
CalAgo exhibits DNA-guided DNA and RNA endonuclease activity at 37 °C. (**A**). Maximum likelihood phylogenetic tree of characterized long-A pAgo proteins. The bootstrap values at the nodes represent the confidence levels from a maximum likelihood analysis based on 1000 resampled data sets. Different colors denote distinct guide–target preferences. Absence of color indicates no detectable cleavage activity under the experimental conditions. Yellow, gDNA-DNA targets; orange, gDNA-DNA targets and RNA targets; dark green, gDNA-DNA targets and RNA targets, gRNA-DNA targets and RNA targets; light green, gDNA-DNA targets and RNA targets, gRNA-DNA targets; purple, gDNA-RNA targets; light blue, gRNA-RNA targets, gDNA-RNA targets; and pink, gDNA-DNA targets, gRNA-DNA targets. (**B**) Guide and target oligonucleotides are shown, with the black triangle marking the cleavage position. (**C**) CalAgo demonstrates DNA-guided endonuclease activity toward both DNA and RNA substrates. The locations of the cleavage products (P) are labeled on the left side of the gels. Reactions were performed using CalAgo, guide, and target at a 6:2:1 molar ratio, incubated at 37 °C for 60 min in the presence of 10 mM Mn^2+^. The catalytically inactive mutant (CalAgo-DM) served as a negative control. Original images can be found at Supplementary Materials.

To identify novel pAgo candidates with potential catalytic activity, we conducted a phylogenetic analysis based on the amino acid sequence of the well-characterized KmAgo using the NCBI database. Among the analyzed homologs, CalAgo was selected as a promising candidate due to its close evolutionary relationship with the mesophilic PbAgo (Figure 1A). Sequence alignment revealed that CalAgo shares 79.01% identity with PbAgo. Moreover, multiple sequence comparisons confirmed the presence of a conserved DEDD catalytic tetrad in CalAgo (Figure S1), suggesting that it retains endonucleolytic activity.

### 3.2. CalAgo Mediates DNA-Guided DNA and RNA Cleavage at 37 °C

The recombinant CalAgo and CalAgo-DM were heterologously expressed in *E. coli* BL21 (DE3), and the soluble fraction was purified and analyzed by SDS-PAGE. The purified protein showed a clear band, consistent with the predicted molecular weight of recombinant CalAgo (82 kDa) (Figure S2). Next, we tested the purified recombinant CalAgo endonuclease activity using an in vitro cleavage assay. We used 18 nt RNA or DNA containing a 5′-phosphorylated (5′P) or 5′-hydroxylated (5′OH) group as guides to cleave complementary 45 nt FAM-labeled single-stranded DNA (ssDNA) or single-stranded RNA (ssRNA) targets, respectively (Figure 1B). After a 60 min incubation at 37 °C, the resulting products were separated on a 20% denaturing polyacrylamide gel. The cleavage assays verified that CalAgo does not show RNA-guided cleavage of targets and only uses DNA guides to cleave DNA and RNA targets (Figure 1C). CalAgo employed 5′P-gDNA to cleave both ssDNA and RNA targets. While DNA cleavage was observed with 5′OH-gDNA, no RNA cleavage was observed with the same guide. The catalytic tetrad in the PIWI domain was essential for CalAgo cleavage, and mutations in this tetrad abolished CalAgo activity. In the absence of the CalAgo protein, no cleavage products were detected.

### 3.3. Effects of Temperature, pH, and Metal Ion on CalAgo Cleavage Activity

**Figure 2:**
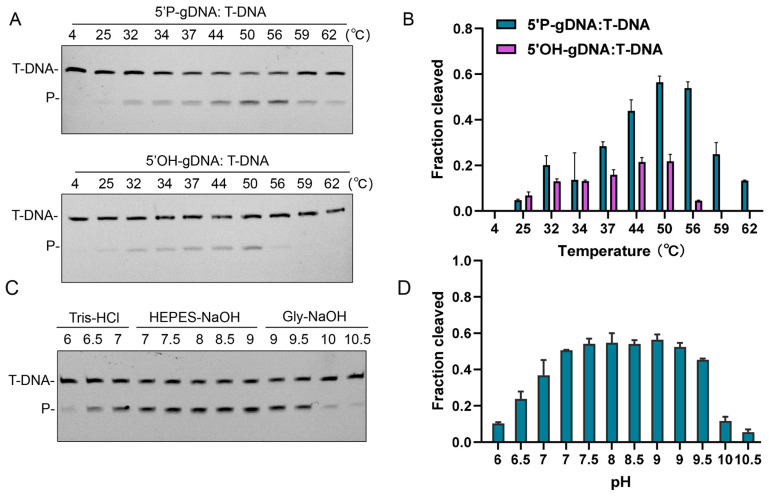
The impact of temperature and pH on the activity of CalAgo. (**A**) Effects of temperature on 5′P-gDNA- (upper panel) and 5′OH-gDNA (lower panel)-mediated DNA cleavage. (**C**) Effects of pH on 5′P-gDNA-mediated DNA cleavage. (**B**,**D**) Quantification of cleavage efficiencies. Data are the mean ± SD from three independent measurements. P, cleavage products. In Figure (**A**,**B**), CalAgo, guide, and target were mixed at a 6:2:1 molar ratio and incubated for 10 min at 37 °C in the presence of 0.5 mM Mn^2+^. In Figure (**C**), the reaction time was 15 min. Original images can be found at Supplementary Materials.

To define the temperature range supporting CalAgo activity, cleavage reactions were conducted between 35 °C and 75 °C (Figure 2A,B). The enzyme displayed distinct optimal ranges depending on the guide type: reactions guided by 5′P-gDNA showed maximal activity at 50–56 °C, whereas those using 5′OH-gDNA maintained relatively constant activity from 44 °C to 50 °C. CalAgo exhibited the highest catalytic efficiency under mildly alkaline conditions (pH 7.0–9.5) (Figure 2C,D).

**Figure 3:**
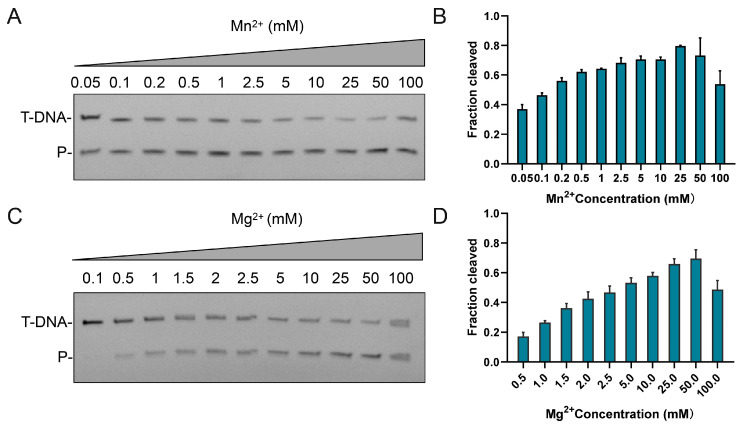
Effects of Me^2+^concentration on CalAgo. (**A**) Effects of Mn^2+^ concentration on 5′P-gDNA-mediated DNA cleavage. (**C**) Effects of Mg^2+^ concentration on 5′P-gDNA-mediated DNA cleavage. P, cleavage products. (**B**,**D**) Quantification of cleavage efficiencies. Data are the mean ± SD from three independent measurements. In all reactions, CalAgo, guide, and target were mixed at a 6:2:1 molar ratio and incubated for 30 min at 37 °C. Original images can be found at Supplementary Materials.

Given that divalent metal ions are crucial for Ago catalysis, we further examined the metal dependence of CalAgo. The enzyme was active with both Mn^2+^ and Mg^2+^ cofactors (Figure S3). Metal titration assays revealed robust activity between 0.5 and 50 mM Mn^2+^ (Figure 3A,B) and at Mg^2+^ concentrations above 0.5 mM (Figure 3C,D). However, elevated levels of either ion (≥100 mM) noticeably suppressed CalAgo-mediated cleavage.

### 3.4. Effects of gDNA Length on Cleavage Activity

**Figure 4:**
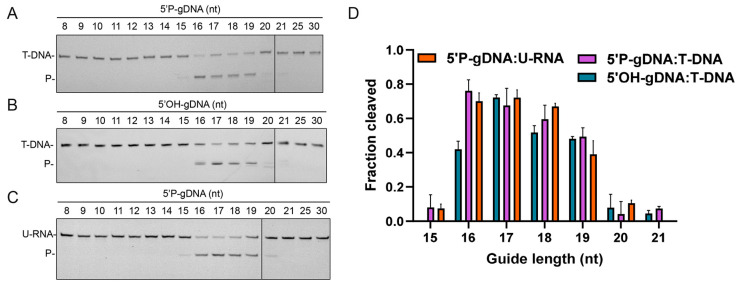
Effects of the guide length on CalAgo activity. (**A**) DNA cleavage assays utilizing 5′P-gDNA of different lengths. (**B**) DNA cleavage assays utilizing 5′OH-gDNA of different lengths. (**C**) RNA cleavage assays utilizing 5′P-gDNA of different lengths. (**D**) Quantification of cleavage efficiencies. Data are the mean ± SD from three independent measurements. P, cleavage products. In all reactions, CalAgo, guide, and target were mixed at a 6:2:1 molar ratio and incubated for 60 min at 37 °C in the presence of 0.5 mM Mn^2+^. Original images can be found at Supplementary Materials.

Previous structural studies revealed that formation of at least a 15 bp guide–target duplex is essential for transitioning from an inactive to a catalytically competent conformation [14]. Here, we examined the minimal guide length required for CalAgo activity. Remarkably, unlike other pAgos that utilize a broad spectrum of guide lengths (15–30 nt) to cleave both DNA and RNA targets [22,27,39], CalAgo displayed activity only with relatively short guides ranging from 15 to 21 nt (Figure 4). The most efficient cleavage occurred with guides of 16–19 nt, whereas longer guides led to a marked decline in catalytic performance (Figure 4). Interestingly, when using a 20 nt 5′OH-gDNA, a slight shift in the cleavage position was observed (Figure 4B), suggesting a subtle effect of the 5′-terminus on cleavage precision.

**Figure 5:**
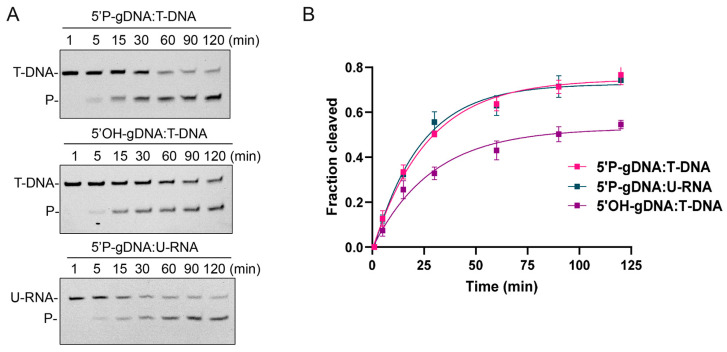
Effects of the 5′-end modification of gDNA on CalAgo-mediated DNA/RNA cleavage activity. (**A**) Cleavage kinetics assays of 5′P-gDNA-mediated DNA cleavage (upper panel), 5′OH-gDNA-mediated DNA cleavage (middle panel), and 5′P-gDNA-mediated RNA cleavage (lower panel). P, cleavage products. (**B**) Quantification of cleavage efficiencies. Data are the mean ± SD from three independent measurements. In all reactions, CalAgo, guide, and target were mixed at a 6:2:1 molar ratio and incubated at 37 °C in the presence of 0.5 mM Mn^2+^. Original images can be found at Supplementary Materials.

In most pAgos, the 5′-end of the guide strand is secured by a conserved sequence motif within the MID domain [7]. The first two amino acids of this motif are highly conserved and serve as the basis for grouping pAgos into several classes, such as YK-, HK-, RK-, and MID-OH-type proteins [6]. Sequence alignment of CalAgo’s MID domain with other well-characterized pAgos revealed that its corresponding residues are Y440 and K444, placing CalAgo within the YK subtype (Figure S4). We next examined the kinetics of CalAgo using 5′P and 5′OH guides in cleavage reactions. CalAgo could use 5′OH-gDNA to cleave the target DNA (0.03493 min^−1^, 95% confidence interval, CI: 0.02514–0.04692 min^−1^) with only slightly lower efficiency in comparison to 5′P-gDNA (0.03784 min^−1^, 95% confidence interval, CI: 0.03146–0.04505 min^−1^) (Figure 5A,B), which is similar to other characterized YK-type pAgos, like CbAgo [21]. In addition, CalAgo was capable of cleaving RNA targets when guided by 5′P-gDNA, with a reaction rate (0.04409 min^−1^, 95% confidence interval, CI: 0.03630–0.05302 min^−1^) comparable to that of DNA cleavage (Figure 5). However, no RNA cleavage activity was detected when 5′OH-gDNA was used as the guide even after a longer reaction time (Figure S5). EMSA further demonstrated that CalAgo could bind DNA in the presence of 5′OH-gDNA but failed to form detectable complexes with RNA (Figure S6). These observations suggest that the lack of RNA cleavage activity under 5′OH-gDNA conditions likely results from CalAgo’s inability to efficiently bind RNA with 5′OH-guided complexes.

### 3.5. Effects of 5′-End Nucleotide and Mismatches of gDNA on Cleavage Activity

**Figure 6:**
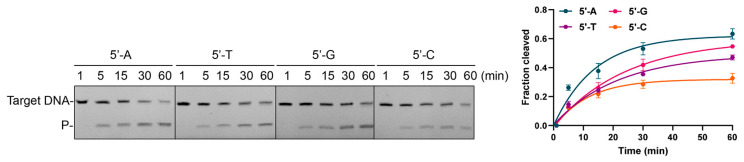
Effects of the 5′-nucleotide of the guide on target cleavage. (**Left panel**) PAGE results, (**right panel**) quantitative results. Data are the mean ± SD from three independent experiments. CalAgo, guide, and target were mixed at a 6:2:1 molar ratio and incubated for different time at 37 °C in the presence of 0.5 mM Mn^2+^. P, cleavage products. Original images can be found at Supplementary Materials.

To assess whether CalAgo exhibits a preference for the 5′-end nucleotide of the guide, we compared the cleavage kinetics using gDNAs beginning with each of the four bases. CalAgo efficiently cleaved ssDNA with all guides, showing only minor variations in reaction rate (Figure 6). The 5′A guide yielded the highest apparent rate constant, whereas 5′G and 5′T guides produced nearly comparable efficiencies, and the 5′C guide showed a slightly reduced rate. Overall, the differences among the four guides were small, indicating that CalAgo’s activity is largely independent of the 5′-end base of the guide. In summary, CalAgo displays no pronounced preference for the 5′-end nucleotide of the guide, with only a marginal decrease in cleavage efficiency observed for the 5′C guide. This suggests that the 5′-base identity exerts minimal influence on CalAgo-mediated catalysis, distinguishing it from Ago proteins that exhibit strong 5′-base specificity [13,40].

**Figure 7:**
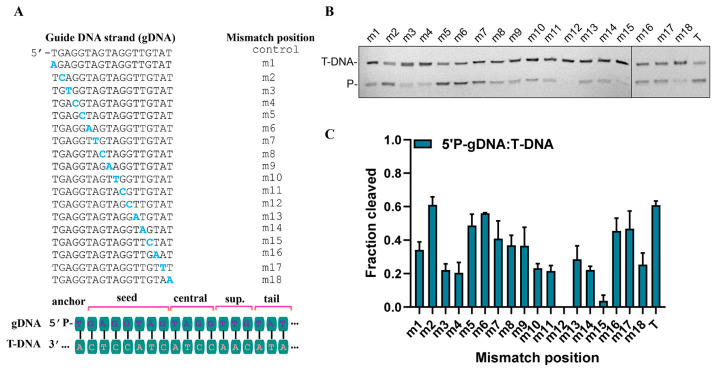
Effects of guide–target mismatches on target cleavage. (**A**) The schematic diagram guide design and mismatch positions. (**B**,**C**) Effects of guide–target mismatches on DNA cleavage by CalAgo. Data are the mean ± SD from three independent measurements. CalAgo, guide, and target were mixed at a 6:2:1 molar ratio and incubated for 30 min at 37 °C in the presence of 0.5 mM Mn^2+^. P, cleavage products. T, control.

Previous reports on pAgos such as MpAgo [23] and CbAgo [21] have indicated that mismatches between guide and target strands can markedly influence substrate recognition and catalytic performance. To assess how single-base mismatches affect CalAgo function, we systematically introduced individual mismatches at positions 1–18 of an 18 nt guide (Figure 7A). CalAgo displayed pronounced sensitivity to mismatches across most positions, except for m2 and m6. In particular, substitutions at positions 12 and 15 completely abolished detectable cleavage activity (Figure 7B,C).

### 3.6. Structure Prediction and Analysis of the PAZ Domain of CalAgo

**Figure 8:**
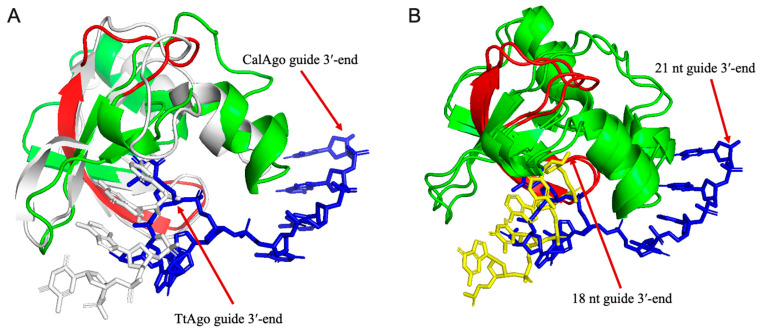
The PAZ domain comparison of the CalAgo with TtAgo via structure prediction. (**A**) Comparison of the CalAgo (green) binary complex carrying a 21 nt guide (blue) with TtAgo binary complex (PDB ID: 3DLH) carrying a 21 nt guide (grey). Only the last seven nucleotides located in the 3′-end of the guide strand were shown. (**B**) Comparison of the CalAgo binary complex (green) carrying a 21 nt guide (blue) with CalAgo binary complex carrying an 18 nt guide (yellow). Only the last four or seven nucleotides located in the 3′-end of the guide strand are shown. Residues D187-V208 in CalAgo are highlighted in red.

Based on the aforementioned findings regarding guide strand binding characteristics, we hypothesize that the distinctive structural conformation of CalAgo may determine its selectivity for guide length and mismatch tolerance, particularly in the interaction mechanism with the 3′-end of gDNA. Previous studies have established that the 3′-end of guide strands is typically anchored within the PAZ domain [12,14]. Through comparative analysis of the crystal structure of TtAgo bound to 21 nt gDNA and the homology model of CalAgo (Figure 8A), we observed that the PAZ domain of TtAgo can stably accommodate the 3′-end of 21 nt gDNA, whereas the homologous domain in CalAgo exhibits significant steric exclusion effects, preventing the 3′-end of gDNA with equivalent length from entering the binding pocket. Further examination of the CalAgo model bound to 18 nt gDNA revealed successful docking of its 3′-end into the nucleic acid-binding pocket (Figure 8B), with this binding site corresponding spatially to the 17th nucleotide position in 21 nt gDNA. These length-dependent conformational differences suggest that CalAgo achieves guide length selection through steric hindrance effects in its PAZ domain.

Notably, studies on *Ferroglobus placidus* Ago (FpAgo) identified that an extra loop in its PAZ domain might result in spatial obstruction, thereby explaining its preference for shorter guide strands [41]. However, our multiple sequence alignment and structural modeling data clearly demonstrate that CalAgo′s PAZ domain lacks such a loop (Figure 8 and Figure S7). To further explore this length-dependent binding pattern, we compared the Alpha Fold 3-predicted CalAgo binary complex structures with guides ranging from 15 nt to 21 nt in length (Figure 8 and Figure S8). The results revealed that only guides of 16–19 nt could be stably accommodated in the PAZ domain, whereas 15 nt, 20 nt, and 21 nt guides failed to anchor their 3′-ends, consistent with the observed cleavage activity profiles. Structural inspection showed that in the CalAgo-15 nt complex, a loop within the N-terminal domain (residues V37–L40) adopts a slightly more compact conformation compared to the corresponding loop (V37–E43) in other complexes, with residue H41 forming a contact with g13. However, this loop reverts to its extended conformation in the 20 nt and 21 nt models, and no other obvious structural elements were identified that could directly explain the inability of very short or long guides to engage the PAZ domain.

Together, these findings suggest that CalAgo’s guide length preference is mainly governed by subtle steric and conformational constraints near the PAZ domain, rather than by a single distinct structural feature such as the extra loop observed in FpAgo [41]. Nevertheless, high-resolution structures of CalAgo in complex with guides of various lengths will be essential to fully elucidate the molecular mechanism underlying its length selectivity.

## 4. Discussion

pAgos exhibit remarkable catalytic diversity, particularly in substrate specificity. While some members exclusively target DNA or RNA, others possess dual DNA/RNA cleavage capabilities [20]. This study characterizes CalAgo, a pAgo derived from the mesophilic bacterium *Cohnella algarum*. Although CalAgo shares high sequence homology with PbAgo, its substrate preference more closely resembles that of KmAgo and TtAgo, which show lower homology. Unlike DNA-specific PbAgo, CalAgo achieves dual targeting of both DNA and RNA using 5′P-gDNA under moderate temperatures, with comparable cleavage activity toward both substrates. Furthermore, when employing 5′OH-gDNA, CalAgo cleaves DNA but loses RNA cleavage functionality. This type of guide-dependent specificity has been observed in other pAgo subtypes, noting that similar characteristics have been reported in some YK-type pAgos such as KmAgo [28].

CalAgo demonstrates broad temperature tolerance (retaining activity at 57 °C) and requires divalent metal ions for catalytic activity, with Mn^2+^ significantly outperforming Mg^2+^. The protein accommodates guides with all four possible 5′-terminal nucleotides with only a marginal decrease in cleavage efficiency observed for the 5′C guide. CalAgo maintains high specificity, where single-nucleotide mismatches in the guide strand substantially impair cleavage activity. Mismatches specifically at positions 12 or 15 completely abolish detectable activity. These combined properties position CalAgo as a promising platform for developing novel nucleic acid detection tools.

Notably, CalAgo′s guide length restriction specificity resembles DNA-targeting pAgos like PbAgo and thermophilic FpAgo, requiring short guides for cleavage activity. In contrast, dual-substrate pAgos such as TtAgo and KmAgo accommodate guides up to 30 nt [27,39]. Structural studies of TtAgo reveal that the retention/release state of the guide strand′s 3′-end within the PAZ domain binding pocket drives the transition of the ternary complex from cleavage-incompatible to cleavage-compatible conformations [14]. For FpAgo, an extra loop insertion in its PAZ domain might sterically hinder accommodation of the 3′-terminus of long guides within the nucleic acid-binding channel, explaining its short-guide dependency. However, our multiple sequence alignments and structural modeling indicate that although CalAgo similarly restricts 3′-end insertion of long guides into its PAZ pocket, this domain lacks such a loop insertion. Future structural determination of CalAgo in binary/ternary complexes will be essential to elucidate the molecular basis of its short-guide dependency.

## 5. Conclusions

In conclusion, this study characterized CalAgo, a pAgo from the mesophilic bacterium *Cohnella algarum*, which exhibits DNA-guided endonuclease activity targeting both ssDNA and RNA at physiological temperatures. CalAgo’s cleavage activity remains stable across a broad range of temperatures and pH, and shows strict specificity for short gDNAs of 15–21 nt in length, in contrast to the broad guide selectivity of other dual DNA/RNA-targeting pAgos. Additionally, CalAgo is highly sensitive to guide–target mismatches, with single mismatches at positions 12 or 15 abolishing detectable activity, consistent with observations in other mesophilic pAgos such as KmAgo [27] and CbAgo [21]. Together, these unique features, including its mesophilic nature, stringent guide length requirement, and mismatch sensitivity, expand our understanding of functional diversity in mesophilic pAgos and highlight CalAgo’s promising potential for broad biotechnological applications. In particular, its ability to function efficiently under physiological conditions enables potential use in programmable nucleic acid detection systems, sequence-specific RNA degradation, and precise regulation of gene expression. Moreover, CalAgo could contribute to the development of RNA interference-like tools, optimization of antisense oligonucleotides, and targeted suppression of viral or mobile genetic elements in mesophilic organisms.

## Data Availability

All relevant data of this study are presented. Additional data will be provided upon request.

## Supplementary Materials

The following supporting information can be downloaded at https://www.mdpi.com/article/10.3390/biom15101459/s1, Figure S1: Multiple sequence alignment of the PIWI domain from CalAgo with several other characterized pAgo proteins; Figure S2: Purification of CalAgo; Figure S3: Effects of divalent cations on CalAgo cleavage activity; Figure S4: Time–course analysis of CalAgo-mediated RNA cleavage using 5′OH-gDNA; Figure S5: Multiple sequence alignment of partial MID domain from CalAgo with several other characterized pAgo proteins; Figure S6: Electrophoretic mobility shift assays (EMSA); Figure S7: Multiple sequence alignment of partial PAZ domain from CalAgo with several other characterized pAgo proteins; Figure S8: Structural comparison of predicted CalAgo binary complexes bound to gDNAs of 15–20 nt in length. Table S1: Oligonucleotides used in cleavage assays; Table S2: The gene sequence of CalAgo; Table S3: Raw data corresponding to Figure 5B; Table S4: Raw data corresponding to Figure 7C.

## References

[1] Swarts D.C., Makarova K., Wang Y., Nakanishi K., Ketting R.F., Koonin E.V., Patel D.J., van der Oost J. (2014). The evolutionary journey of Argonaute proteins. Nat. Struct. Mol. Biol..

[2] Koonin E.V. (2017). Evolution of RNA- and DNA-guided antivirus defense systems in prokaryotes and eukaryotes: Common ancestry vs convergence. Biol. Direct.

[3] Shabalina S.A., Koonin E.V. (2008). Origins and evolution of eukaryotic RNA interference. Trends Ecol. Evol..

[4] Bartel D.P. (2009). MicroRNAs: Target recognition and regulatory functions. Cell.

[5] Kuzmenko A., Oguienko A., Esyunina D., Yudin D., Petrova M., Kudinova A., Maslova O., Ninova M., Ryazansky S., Leach D. (2020). DNA targeting and interference by a bacterial Argonaute nuclease. Nature.

[6] Ryazansky S., Kulbachinskiy A., Aravin A.A. (2018). The Expanded Universe of Prokaryotic Argonaute Proteins. mBio.

[7] Lisitskaya L., Aravin A.A., Kulbachinskiy A. (2018). DNA interference and beyond: Structure and functions of prokaryotic Argonaute proteins. Nat. Commun..

[8] Loeff L., Adams D.W., Chanez C., Stutzmann S., Righi L., Blokesch M., Jinek M. (2024). Molecular mechanism of plasmid elimination by the DdmDE defense system. Science.

[9] Hegge J.W., Swarts D.C., van der Oost J. (2018). Prokaryotic Argonaute proteins: Novel genome-editing tools?. Nat. Rev. Microbiol..

[10] Jin S., Zhan J., Zhou Y. (2021). Argonaute proteins: Structures and their endonuclease activity. Mol. Biol. Rep..

[11] Koopal B., Mutte S.K., Swarts D.C. (2023). A long look at short prokaryotic Argonautes. Trends Cell Biol..

[12] Tao X., Ding H., Wu S., Wang F., Xu H., Li J., Zhai C., Li S., Chen K., Wu S. (2024). Structural and mechanistic insights into a mesophilic prokaryotic Argonaute. Nucleic Acids Res..

[13] Nakanishi K., Weinberg D.E., Bartel D.P., Patel D.J. (2012). Structure of yeast Argonaute with guide RNA. Nature.

[14] Sheng G., Zhao H., Wang J., Rao Y., Tian W., Swarts D.C., van der Oost J., Patel D.J., Wang Y. (2014). Structure-based cleavage mechanism of Thermus thermophilus Argonaute DNA guide strand-mediated DNA target cleavage. Proc. Natl. Acad. Sci. USA.

[15] Miyoshi T., Ito K., Murakami R., Uchiumi T. (2016). Structural basis for the recognition of guide RNA and target DNA heteroduplex by Argonaute. Nat. Commun..

[16] Swarts D.C., Hegge J.W., Hinojo I., Shiimori M., Ellis M.A., Dumrongkulraksa J., Terns R.M., Terns M.P., van der Oost J. (2015). Argonaute of the archaeon *Pyrococcus furiosus* is a DNA-guided nuclease that targets cognate DNA. Nucleic Acids Res..

[17] Yuan Y.R., Pei Y., Ma J.B., Kuryavyi V., Zhadina M., Meister G., Chen H.Y., Dauter Z., Tuschl T., Patel D.J. (2005). Crystal structure of *A. aeolicus* argonaute, a site-specific DNA-guided endoribonuclease, provides insights into RISC-mediated mRNA cleavage. Mol. Cell.

[18] Swarts D.C., Jore M.M., Westra E.R., Zhu Y., Janssen J.H., Snijders A.P., Wang Y., Patel D.J., Berenguer J., Brouns S.J.J. (2014). DNA-guided DNA interference by a prokaryotic Argonaute. Nature.

[19] Zander A., Willkomm S., Ofer S., van Wolferen M., Egert L., Buchmeier S., Stockl S., Tinnefeld P., Schneider S., Klingl A. (2017). Guide-independent DNA cleavage by archaeal Argonaute from *Methanocaldococcus jannaschii*. Nat. Microbiol..

[20] Graver B.A., Chakravarty N., Solomon K.V. (2024). Prokaryotic Argonautes for in vivo biotechnology and molecular diagnostics. Trends Biotechnol..

[21] Kuzmenko A., Yudin D., Ryazansky S., Kulbachinskiy A., Aravin A.A. (2019). Programmable DNA cleavage by Ago nucleases from mesophilic bacteria *Clostridium butyricum* and *Limnothrix rosea*. Nucleic Acids Res..

[22] Jiang X., Liu Y., Liu Q., Ma L. (2022). Characterization of a Programmable Argonaute Nuclease from the Mesophilic Bacterium *Rummeliibacillus suwonensis*. Biomolecules.

[23] Kaya E., Doxzen K.W., Knoll K.R., Wilson R.C., Strutt S.C., Kranzusch P.J., Doudna J.A. (2016). A bacterial Argonaute with noncanonical guide RNA specificity. Proc. Natl. Acad. Sci. USA.

[24] Bastiaanssen C., Bobadilla Ugarte P., Kim K., Finocchio G., Feng Y., Anzelon T.A., Kostlbacher S., Tamarit D., Ettema T.J.G., Jinek M. (2024). RNA-guided RNA silencing by an Asgard archaeal Argonaute. Nat. Commun..

[25] Li W., Liu Y., He R., Wang L., Wang Y., Zeng W., Zhang Z., Wang F., Ma L. (2022). A programmable pAgo nuclease with RNA target preference from the psychrotolerant bacterium *Mucilaginibacter paludis*. Nucleic Acids Res..

[26] Lisitskaya L., Shin Y., Agapov A., Olina A., Kropocheva E., Ryazansky S., Aravin A.A., Esyunina D., Murakami K.S., Kulbachinskiy A. (2022). Programmable RNA targeting by bacterial Argonaute nucleases with unconventional guide binding and cleavage specificity. Nat. Commun..

[27] Liu Y., Li W., Jiang X., Wang Y., Zhang Z., Liu Q., He R., Chen Q., Yang J., Wang L. (2021). A programmable omnipotent Argonaute nuclease from mesophilic bacteria *Kurthia massiliensis*. Nucleic Acids Res..

[28] Kropocheva E., Kuzmenko A., Aravin A.A., Esyunina D., Kulbachinskiy A. (2021). A programmable pAgo nuclease with universal guide and target specificity from the mesophilic bacterium *Kurthia massiliensis*. Nucleic Acids Res..

[29] Li Y., Zhao L., Wang J., Ma L., Bai Y., Feng F. (2024). Argonaute-Based Nucleic Acid Detection Technology: Advantages, Current Status, Challenges, and Perspectives. ACS Sens..

[30] Enghiad B., Xue P., Singh N., Boob A.G., Shi C., Petrov V.A., Liu R., Peri S.S., Lane S.T., Gaither E.D. (2022). PlasmidMaker is a versatile, automated, and high throughput end-to-end platform for plasmid construction. Nat. Commun..

[31] Esyunina D., Okhtienko A., Olina A., Panteleev V., Prostova M., Aravin A.A., Kulbachinskiy A. (2023). Specific targeting of plasmids with Argonaute enables genome editing. Nucleic Acids Res..

[32] Jinek M., Chylinski K., Fonfara I., Hauer M., Doudna J.A., Charpentier E. (2012). A programmable dual-RNA-guided DNA endonuclease in adaptive bacterial immunity. Science.

[33] Vaiskunaite R., Vainauskas J., Morris J.J.L., Potapov V., Bitinaite J. (2022). Programmable cleavage of linear double-stranded DNA by combined action of Argonaute CbAgo from Clostridium butyricum and nuclease deficient RecBC helicase from *E. coli*. Nucleic Acids Res..

[34] Liu Y., Zhang J., Liu J., Zhang S., An L., Xie W., Zhang K., Li S. (2025). The PAZ pocket and dimerization drive CpAgo’s guide-independent and DNA-guided dual catalysis. Nat. Commun..

[35] Swarts D.C., Szczepaniak M., Sheng G., Chandradoss S.D., Zhu Y., Timmers E.M., Zhang Y., Zhao H., Lou J., Wang Y. (2017). Autonomous Generation and Loading of DNA Guides by Bacterial Argonaute. Mol. Cell.

[36] Kumar S., Stecher G., Li M., Knyaz C., Tamura K. (2018). MEGA X: Molecular Evolutionary Genetics Analysis across Computing Platforms. Mol. Biol. Evol..

[37] Yu F., Li X., Wang F., Liu Y., Zhai C., Li W., Ma L., Chen W. (2023). TLTC, a T5 exonuclease-mediated low-temperature DNA cloning method. Front. Bioeng. Biotechnol..

[38] Abramson J., Adler J., Dunger J., Evans R., Green T., Pritzel A., Ronneberger O., Willmore L., Ballard A.J., Bambrick J. (2024). Accurate structure prediction of biomolecular interactions with AlphaFold 3. Nature.

[39] Wang Y., Juranek S., Li H., Sheng G., Tuschl T., Patel D.J. (2008). Structure of an argonaute silencing complex with a seed-containing guide DNA and target RNA duplex. Nature.

[40] Willkomm S., Oellig C.A., Zander A., Restle T., Keegan R., Grohmann D., Schneider S. (2017). Structural and mechanistic insights into an archaeal DNA-guided Argonaute protein. Nat. Microbiol..

[41] Guo X., Sun Y., Chen L., Huang F., Liu Q., Feng Y. (2021). A Hyperthermophilic Argonaute from *Ferroglobus placidus* with Specificity on Guide Binding Pattern. Front. Microbiol..

