# Supplementary material for: A Mesophilic Argonaute from Cohnella algarum Mediates Programmable DNA/RNA Cleavage with Distinctive Guide Specificity
Source: Biomolecules. 2025 Oct 16;15(10):1459. doi: 10.3390/biom15101459 (PMC12564509; doi:10.3390/biom15101459)
Supplement: Supplementary file 1 [file biomolecules-15-01459-s001.zip › biomolecules-3855728-supplementary-final-no tc.pdf]

# Supplementary materials

```
KmAgo    522  CFIGIDVSHENGNSAAGMMNVIGSQGHLIQQAPLNGILAGEKIDDTLLANLLKQMIKAYHTQFQRFPKHITIHRDGFWREHTALVEKIM   610
MeAgo    524  CFIGLDVSHEDGKSAAGIMNVIGCNGHLIKQTAINGVLAGEKIDNETLKEIMVDILGSYKQQFGDFPKHVTIHRDGFWREDSKLVDQLL   612
DeAgo    524  CFIGLDVSHEDGKSAAGIMNVIGSNGHLIKQSALNGVLAGEKIDEPTLNEIITEVLYSYQKQFGHFPKHVTIHRDGKWRESSELVGRLF   612
RsuAgo   525  CFIGLDVSHEDGKSTAGIMNVIGSNGYLLKQSAINGVLAGEKIDTEKLEEIIQDVIFTYKEQFGKLPRHVTIHRDGRWREDSVLVAQIF   613
BsAgo    524  CFIGLDVSHEDGKSTAGIMNVIGSNGHLLKQSAIHGALAGEKIDTKTLEEILMDVLFTYKNQFGDFPKHVTIHRDGKWNEDTEFVDKLF   612
IbAgo    512  CYIGLDVCRENNMSTAGLIQVIGKDGRVLKSKTISSHQSGEKIQINILKDIIFEAKQAYKNTYNKKLEHIVFHRDGINREDIDLLKEIT   600
EmaAgo   489  CFVGLDVSHENGKHAAGIIQIIGKDGAMIKQKALSTSEAGEKISSETMREIVYDTLHAFEEQYGHAPKHITFHRDGFGREDLTLIDSIL   577
EsAgo    490  CFVGLDVSHENGKHASGIIQIIGKDGAMIKQKSLSTSEAGEKISAETMREIVYDTLHAFEEQYDHAPMHITFHRDGFCREDLALIDSLL   578
CalAgo   497  CFIGLDVSHESGRHSAGIVQIVGKDGRVLSSRANTSNEAGEKIRHETMCQIIYSAIDQYQRHYNEKPRHVTFHRDGLCREDLNSLDEVF   585
PbAgo    493  CFIGLDVSHEAGRHSTGIVQVVGKDGRVLSSKANTSNEAGEKIRHETMCQIVYSAIDQYQQHYNERPKHVTFHRDGFCREDLLSLDEVM   581
BlAgo    487  CFIGLDVSHEENRHSTGLVQVVGKDGRVLSSKAMSTIESGERIRDETMKEIVYEAIHSYENQYGYRPKHVTFHRDGFCRENIDNIEYIL   575

                                                 *
KmAgo    680  LPFSHIIEDVYNLSFLHIHAMNKMRLPATIHYADLSATAYQRGQVMPRSGNQTNLPFV   737
MeAgo    686  LSINQIIEDVYRLSFMHIHALNKMRLPATVHYADLSSTAYQRGQVSPRSTNLTHLPFV   743
DeAgo    682  LPFKQIIQDCYDLSFMHIHAVNKMRLPATIHYADLSSTAYQRGQVAPRTTNGTHLPFV   739
RsuAgo   683  LAFQQIIEDIYRLSFMHIHALNKMRLPATIHYADLSSTAYQRGQIAPRTTNVTHLPFV   740
BsAgo    682  LTFEQLIEDVYRLSFMHIHSLNKMRLPATIHYADLSSTAYQRGQVAPRTTNVTHLPFV   739
IbAgo    680  QNMDDIVKDIYKLSFMHIGSIMKSRLPITTYYADLSSIYSHRE-LMPKSVDNNILHFI   736
EmaAgo   644  LPFEAILTDVYRLSFMHVHSLLKTRLPITTHYADLSSTFHNRGLLNANTEHEEALPFV   701
EsAgo    645  LKFEDILSDVYRLSFMHVHSLLKTRLPITTHYADLSSTFHNRGLINANTQHEEALPFV   702
CalAgo   654  LSLEAIVQDIYHLSFMHIGSLLKCRLPITTYYADLSSTFFNRQ--WIPIDSGESLHFV   709
PbAgo    650  LPIEAIIQDIYHLSFMHIGSLLKCRLPITTYYADLSSTFFNRQ--WLPIDSGEALHFV   705
BlAgo    645  LTMSAIVSDIFNLSHMHVGSLIKSRLPISTYYADLSSTFFNRG--WI-SSRSNGLQFV   699
```

**Figure S1. Multiple sequence alignment of the PIWI domain from CalAgo with several other characterized pAgo proteins.** Black asterisks are the positions of catalytic tetrad amino acid residues.

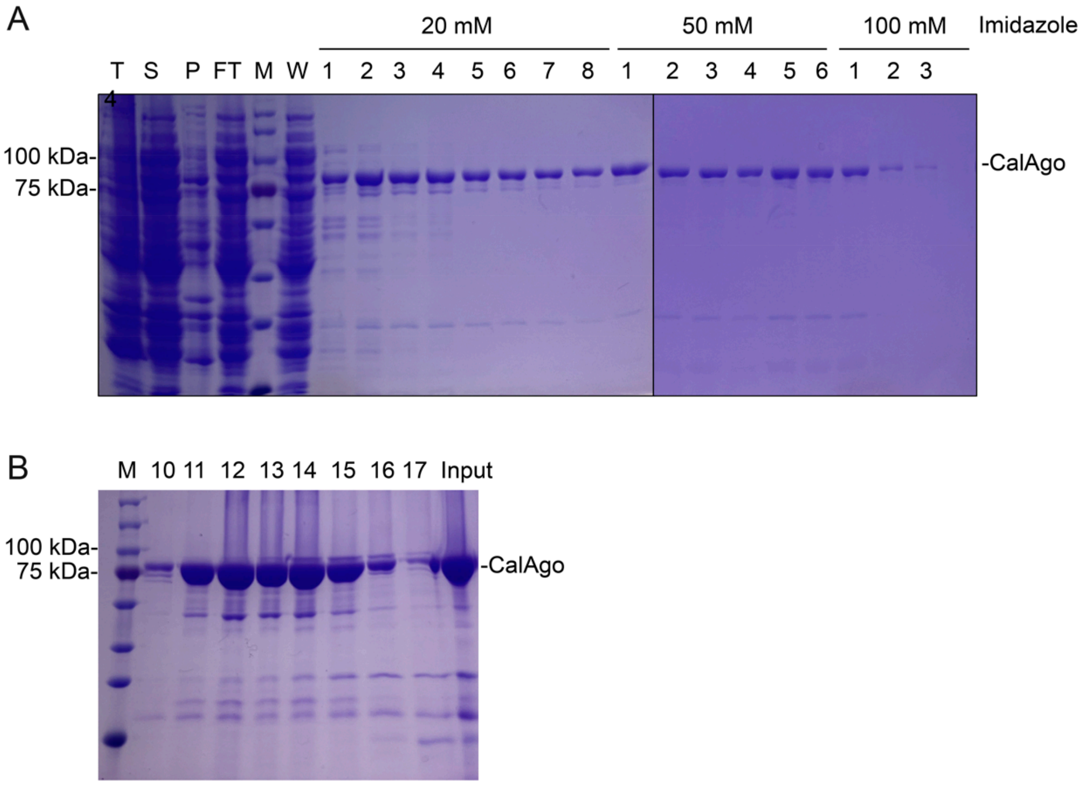


**Figure S2. Purification of CalAgo.** (A) Ni-NTA purification of CalAgo. Lanes: M, molecular weight marker; T, total cell protein fraction; S, soluble fraction; P, insoluble fraction; FT, flow through fraction; W, Buffer A wash fraction. (B) Heparin purification of CalAgo. Input, Ni-NTA purified protein fraction. 10-17, NaCl eluted CalAgo.

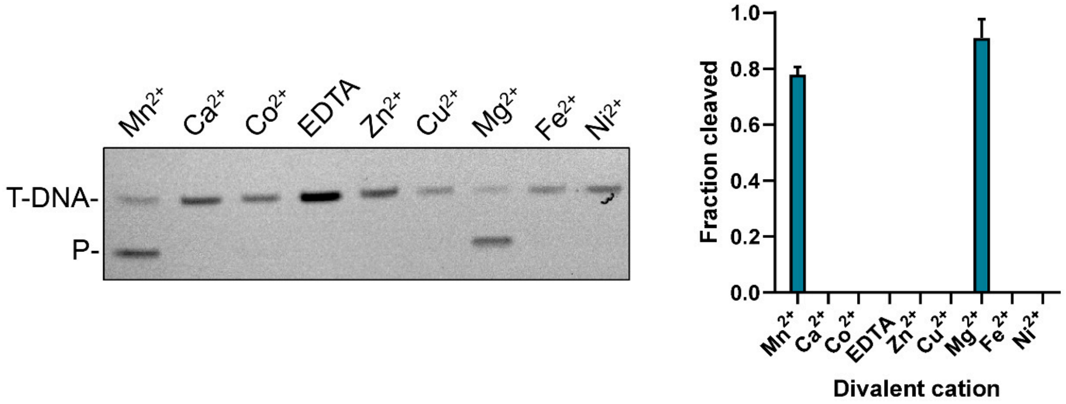


**Figure S3. Effects of divalent cations on CalAgo cleavage activity.** Data are the mean ± SD from three independent measurements. CalAgo, guide and target were mixed at a 6:2:1 molar ratio and incubated for 60 min at 37 °C in the presence of 0.5 mM $Mn^{2+}$.

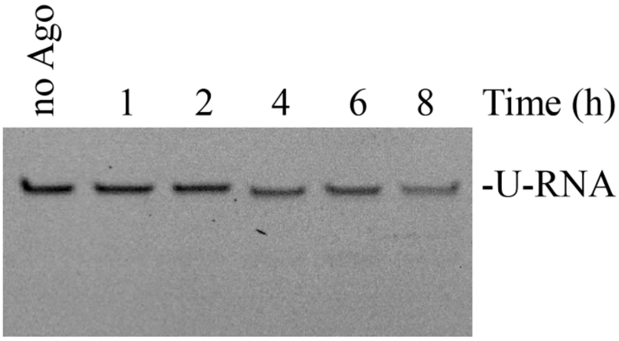


**Figure S4. Time-course analysis of CalAgo-mediated RNA cleavage using 5′ OH-gDNA.**

```
                        Y   K           Q                                                        K
FpAgo     490   EGEDSFDYYNPLKSAL-FRNNILSQNFDVTNYVRGDGKINKNTIK------------------------YAVSNIIYNIFGKL  547
KmAgo     429   KKTRS---YQLLKQYFGGKWDIASQVITEK-TIEAFQKILHKHGLKNFYPNDEQHCLRVIDVLKNESFYYTVMNILLGVYVKS  507
MeAgo     431   MPIKI---YSEFKRQFGGKWDLSSQVLTEK-SIQSFKNILKQHKLEDFNVHDNLACQKVANTVKNSALSFTIFNILLGLYVKS  509
DeAgo     431   LPNKL---YNEFKRQFGGKWDISSQIITEK-VLKTFQYALTRHQLTDFNVNDEKECERVADIVKNDSLSYPVFNILLGIYVKS  509
RsuAgo    432   LPVKV---YDAFKRQFGGKWDISSQVITEK-SLKSFQQLLKIRKLEEFDLNDEKLCQQVANIVKYDNLSYTIFNILLGIYVKS  510
BsAgo     431   LPIKV---YDAFKRQFGGKWDISTQVITEN-SLKLFQQLLKRNQLDLFNPNDEVVCQEVANRIKSDNLFYTINNILLGVYVKS  509
IbAgo     445   Y-------YETLKKIFGGRNNIPTQFVDLD-TIKKCDPKIDNKRGKE----------------------SIFLNILLGIYCKS  497
EmaAgo    431   S-------YQAIKRQFGGKQDVVTQCVELHDRVLNSE--------------------------------DTLYNILLGIYVKA  474
EsAgo     432   S-------YQAIKREFGGKHDVVTQCVELHDRVLNSE--------------------------------DTLYNILLGVYVKA  475
CalAgo    439   W-------YDLIKKEFGGNSSVPTQFITIE-TLQKAND-------------------------------FILGNLLLGLYSKS  482
PbAgo     435   W-------YDLVKKEFGGNSSVPTQFITIE-TLQKAND-------------------------------YILGNLLLGLYSKS  478
BlAgo     429   C-------YGAIKKEFGGNYDIPTQFVTAD-TAKEKND-------------------------------YILLNILLGIYAKA  472
```

**Figure S5. Multiple sequence alignment of partial MID domain from CalAgo with several other characterized pAgo proteins.** YKQK motif highlighted in yellow.

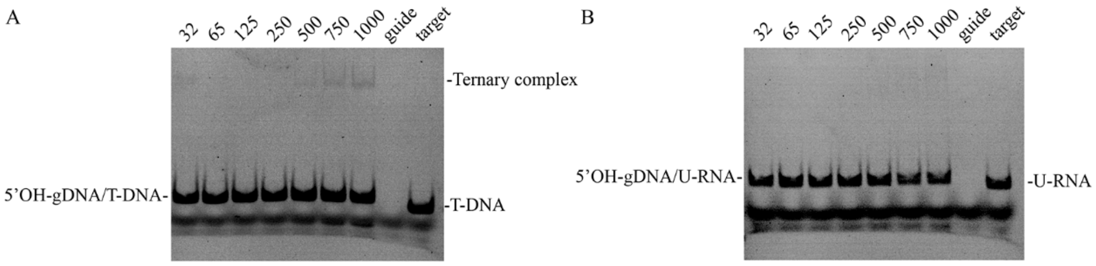


**Figure S6. Electrophoretic mobility shift assays (EMSA).** (A) Binding of CalAgo-DM to the 5′ OH-gDNA/T-DNA duplex. (B) Binding of CalAgo-DM to the 5′ OH-gDNA/U-RNA duplex.

```
FpAgo    179   NDNRIW---FRYIPHLIIEE----VEDFEESAKSF   206
KmAgo    175   RVVHSN---GRQHYTYTVEN----VATYGVTDRCP   202
MeAgo    176   KIIHSD---PTTNFTYEFDR----IADYSVMDICP   203
DeAgo    176   KVVHSD---VWNNYTYEVEQ----IAPYSVMDMCP   203
RsuAgo   176   KVVHSD---VRNNYTYEVEQ----VAPYSVMETCQ   203
BsAgo    176   KVLHSD---IRNNYTYEVEQ----IAPYSVMEYCP   203
IbAgo    193   KVKDFY-----NNITYEFLE----MAPFSISQENK   218
EmaAgo   179   RVIDST---HPKSYEYEFVE----VAPYRANEVSP   206
EsAgo    179   RVIDST---HPKSYEYEFVE----VAPYRASEVSP   206
CalAgo   184   RVIDKY-----NNLHYEFDK----VTEYTISDVVP   209
PbAgo    181   RVIDIY-----NNLHYEFVE----ISNSTINDSIP   206
BlAgo    177   RVIDPI-----YHISYVYDE----VAPYTVSESSP   202
TtAgo    192   RVRNAY-----DRRTWELLR----LGEEDPKELPL   217
```

**Figure S7. Multiple sequence alignment of partial PAZ domain from CalAgo with several other characterized pAgo proteins.** Special residues of FpAgo and CalAgo highlighted in yellow.

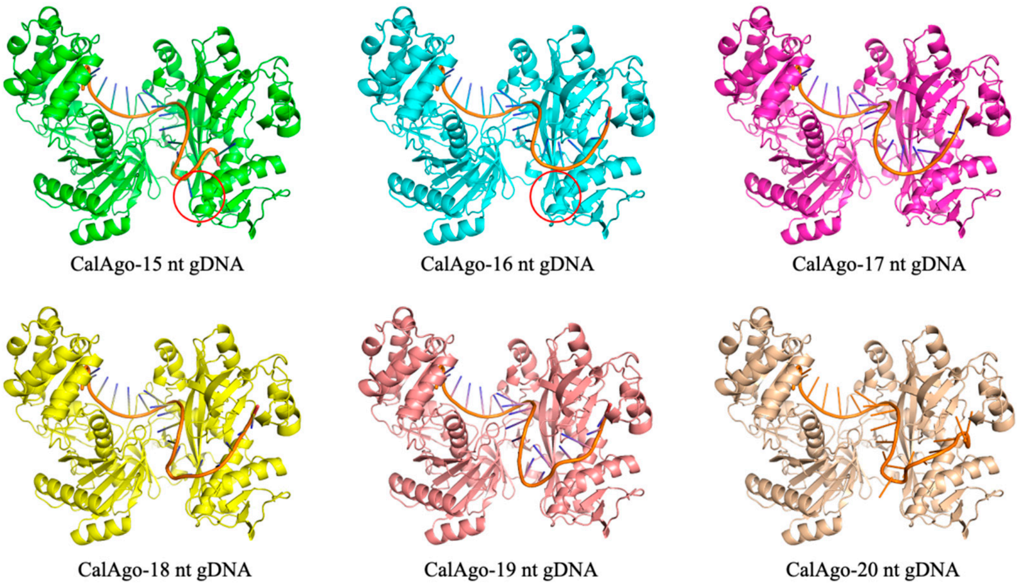


**Figure S8. Structural comparison of predicted CalAgo binary complexes bound to gDNAs of 15-20 nt in length.** The red circle highlights the structural differences in the N-terminal domain between CalAgo bound to 15 nt and 16 nt gDNAs.

**Table S1. Oligonucleotides used in cleavage assays**

| Name | Sequence (5’-3’) | Description |
|---|---|---|
| FAM-T-DNA | FAM-AAACGACGGCCAGTGCCAAGC TTACTATACAACCTACTACCTCAT | Figure 1C, 2, 3, 4, 5, 6, Supplementary Figure 3 |
| FAM-U-RNA | FAM-AAACGACGGCCAGUGCCAAGC UUACUAUACAACCUACUACCUCAU | Figure 1C, 4C, 4D |
| C-gDNA | CGAGGTAGTAGGTTGTAT | Figure 6A |
| T-gDNA | TGAGGTAGTAGGTTGTAT | Figure 1C, 2, 3, 4, 5, 6, Supplementary Figure 3 |
| A-gDNA | AGAGGTAGTAGGTTGTAT | Figure 6A |
| G-gDNA | GGAGGTAGTAGGTTGTAT | Figure 6A |
| U-gRNA | UGAGGUAGUAGGUUGUAU | Figure 1C |
| FAM-A-DNA | FAM-AAACGACGGCCAGTGCCAAGC TTACTATACAACCTACTACCTCTT | Figure 6A |
| FAM-G-DNA | FAM-AAACGACGGCCAGTGCCAAGC TTACTATACAACCTACTACCTCCT | Figure 6A |
| FAM-C-DNA | FAM-AAACGACGGCCAGTGCCAAGC TTACTATACAACCTACTACCTCGT | Figure 6A |
| gDNA_m1 | AGAGGTAGTAGGTTGTAT | Figure 6B |
| gDNA_m2 | TCAGGTAGTAGGTTGTAT | |
| gDNA_m3 | TGTGGTAGTAGGTTGTAT | |
| gDNA_m4 | TGACGTAGTAGGTTGTAT | |
| gDNA_m5 | TGAGCTAGTAGGTTGTAT | |
| gDNA_m6 | TGAGGAAGTAGGTTGTAT | |
| gDNA_m7 | TGAGGTTGTAGGTTGTAT | |
| gDNA_m8 | TGAGGTACTAGGTTGTAT | |
| gDNA_m9 | TGAGGTAGAAGGTTGTAT | |
| gDNA_m10 | TGAGGTAGTTGGTTGTAT | |
| gDNA_m11 | TGAGGTAGTACGTTGTAT | |
| gDNA_m12 | TGAGGTAGTAGCTTGTAT | |
| gDNA_m13 | TGAGGTAGTAGGATGTAT | |

| | | |
|---|---|---|
| gDNA_m14 | TGAGGTAGTAGGTAGTAT | |
| gDNA_m15 | TGAGGTAGTAGGTTCTAT | |
| gDNA_m16 | TGAGGTAGTAGGTTGAAT | |
| gDNA_m17 | TGAGGTAGTAGGTTGTTT | |
| gDNA_m18 | TGAGGTAGTAGGTTGTAA | |
| 8nt T-gDNA | TGAGGTAG | Figure 4 |
| 9nt T-gDNA | TGAGGTAGT | |
| 10nt T-gDNA | TGAGGTAGTA | |
| 11nt T-gDNA | TGAGGTAGTAG | |
| 12nt T-gDNA | TGAGGTAGTAGG | |
| 13nt T-gDNA | TGAGGTAGTAGGT | |
| 14nt T-gDNA | TGAGGTAGTAGGTT | |
| 15nt T-gDNA | TGAGGTAGTAGGTTG | |
| 16nt T-gDNA | TGAGGTAGTAGGTTGT | |
| 17nt T-gDNA | TGAGGTAGTAGGTTGTA | |
| 19nt T-gDNA | TGAGGTAGTAGGTTGTATA | |
| 20nt T-gDNA | TGAGGTAGTAGGTTGTATAG | |
| 21nt T-gDNA | TGAGGTAGTAGGTTGTATAGT | |
| 25nt T-gDNA | TGAGGTAGTAGGTTGTATAGTAAGC | |
| 30nt T-gDNA | TGAGGTAGTAGGTTGTATAGTAAGCT TGGC | |

**Table S2. The gene sequence of CalAgo**

atgttccacatgaataccccgaccacccaccacattctgaccgaatgggaaagcgacaccaacgcaagcaacctggtcgt
tcacctgtacagcctgcccgttcgcaacgtttttgaattacataacgaaaacggccatgcctgcagcgaactgcgtcgcctg
aataaaaccaacgtcatcgatttttcgaacagtttattgccagctggcaacctattgaaaactggggcaaatatacctttacca
atcatgaatgtcggagcataaatgcattagttagcaccgaacgcaccgttctggaacgtctgctgctgcgtaccattgaaag
cgcacagccgcgcaatgaaatcgccgcaggtagccgcaaatttacctggttaaaagccgaaaaagtagtacagaatatca
gcattcatagagttatccattgtgatgttagcattaacattaacggtgttatcacagttggttttgatctgaatcatagcttccgga
ccaacgatagcgtttttgatttaatgaaatcgaaggcaatcatcaagggtgatcgtgtgattgacaaatataataatctgcacta
cgagtttgacaaagttaccgaatataccatcagcgatgttgttccggaactgggtcagagcgttatcaattactttgtgcgtga
aagaaagcagacatggaaagtggacaaactggaccctagcatgccggttgtttatgtgaaaatgaaaaatcgtcaggcac
cgattgcatatgcaccggccatgctgcagaaagaactgacatttgaaagcctgccggtggcagtggttagacagacaagc
gaagtttataaacagaacgccaatcagaaaattaagatactgctggatgaaatgcagaacatcttaaaacggaccgataaat
tacagtttagcaaacaaaagctgctggtacagcagagcgggtacaaagttaaacaaatcgaaatcccgaatctgacctttgg
aaaaaatgttacccagagccagccgcgttatggtctggaaaaaggtggtgttgttctgagcaaaccgctgagcattaatctg
ctggtttatccggaactgattaataccaaactgaatgcaattaccgcatttaatgataaactgattgcactgagccataaatgg
ggtgttccgctgaccattctgaaaaaaagcgaaacctatcgtaataaaagcattgattttaccaatccgcatcagtttgcaattc
tgctgaaagaactggcaaattatagctttcaggaactgaccctggttattattccggaaaaagttgcaggtatgtggtatgatct
gattaaaaaagaatttggtggtaatagcagcgttccgacccagtttattaccattgaaaccctgcagaaagcaaatgattttatt
ctgggtaatctgctgctgggtctgtatagcaaaagcggtattcagccgtggattctgaatagcagcctgagcagcgattgtttt
attggtctggatgttagccatgaaagcggtcgtcatagcgcaggtattgttcagattgttggtaaagatggtcgtgttctgagc
agccgtgcaaataccagcaatgaagcaggtgaaaaaattcgtcatgaaaccatgtgtcagattatttatagcgcaattgatca
gtatcagcgtcattataatgaaaaaccgcgtcatgttacctttcatcgtgatggtctgtgtcgtgaagatctgaatagcctggat
gaagtttttgatagcctgggtgttgattatgatatggttgaaattattaaaaaaaccaatcgtcgtatggcaattaatgttgataaa
cagggttgggaaaccaaaagcggtctgtgttatgttaaagataattgggcatatctgattagcaccaatccgcatccgcgtgt
tggtaccgcacagccgattaaaattgttaaaaaacagggtagcctgagcctggaagcaattgttcaggatatttatcatctga
gctttatgcatattggtagcctgctgaaatgtcgtctgccgattaccacctattatgcagatctgagcagcaccttttttaatcgtc
agtggattccgattgatagcggtgaaagcctgcattttgttctcgag

Table S3. Raw data corresponding to Figure 5B

| Time (min) | 5'P-gDNA:T-DNA | | | 5'OH-gDNA:T-DNA | | |
|---|---|---|---|---|---|---|
| 1 | 0 | 0 | 0 | 0 | 0 | 0 |
| 5 | 0.11687533 | 0.098155964 | 0.165701977 | 0.07920257 | 0.046387345 | 0.095594421 |
| 15 | 0.334943565 | 0.302794947 | 0.365089986 | 0.220622589 | 0.247207465 | 0.299405339 |
| 30 | 0.509533235 | 0.488257812 | 0.512991566 | 0.297643479 | 0.335609447 | 0.351698347 |
| 60 | 0.608878566 | 0.66946797 | 0.632404041 | 0.450930487 | 0.382272326 | 0.458688329 |
| 90 | 0.72944673 | 0.730577478 | 0.678899525 | 0.463731874 | 0.519417439 | 0.524060052 |
| 120 | 0.771682584 | 0.806306138 | 0.720172255 | 0.527936539 | 0.565933768 | 0.542515887 |

| Time (min) | 5'P-gDNA:U-RNA | | |
|---|---|---|---|
| 1 | 0 | 0 | 0 |
| 5 | 0.143679569 | 0.124069162 | 0.107680266 |
| 15 | 0.329987184 | 0.299969752 | 0.340314594 |
| 30 | 0.527132067 | 0.609344282 | 0.531995768 |
| 60 | 0.642778602 | 0.665620666 | 0.580519314 |
| 90 | 0.76459604 | 0.708916395 | 0.668566727 |
| 120 | 0.757015131 | 0.73644963 | 0.736529693 |

Table S4. Raw data corresponding to Figure 7C

| Mismatch position | **5'P-gDNA:T-DNA** | | |
|---|---|---|---|
| m1 | 0.292716009 | 0.388783846 | 0.341938319 |
| m2 | 0.557262828 | 0.652607323 | 0.621298276 |
| m3 | 0.196188238 | 0.263207195 | 0.203154573 |
| m4 | 0.184949574 | 0.273563189 | 0.154513911 |
| m5 | 0.557452237 | 0.424103813 | 0.480857943 |
| m6 | 0.558123785 | 0.563841144 | 0.559356142 |
| m7 | 0.436027858 | 0.292984851 | 0.498451772 |
| m8 | 0.299088271 | 0.402293829 | 0.404757455 |
| m9 | 0.298897592 | 0.492857034 | 0.307031163 |
| m10 | 0.236810088 | 0.257440386 | 0.202920097 |
| m11 | 0.252575442 | 0.190600716 | 0.203056927 |
| m12 | 0 | 0 | 0 |
| m13 | 0.22873223 | 0.250913104 | 0.377024789 |
| m14 | 0.198532002 | 0.240110745 | 0.228196194 |
| m15 | 0.066364927 | 0 | 0.045243163 |
| m16 | 0.39269396 | 0.430846004 | 0.540621674 |
| m17 | 0.388464714 | 0.426677165 | 0.58803004 |
| m18 | 0.231987645 | 0.19816561 | 0.331327812 |
| T | 0.579416583 | 0.628338119 | 0.617508101 |
