# Supplementary material for: A Mesophilic Argonaute from Cohnella algarum Mediates Programmable DNA/RNA Cleavage with Distinctive Guide Specificity
Source: Biomolecules. 2025 Oct 16;15(10):1459. doi: 10.3390/biom15101459 (PMC12564509; doi:10.3390/biom15101459)
Supplement: Supplementary file 1 [file biomolecules-15-01459-s001.zip › Original gels.pdf]

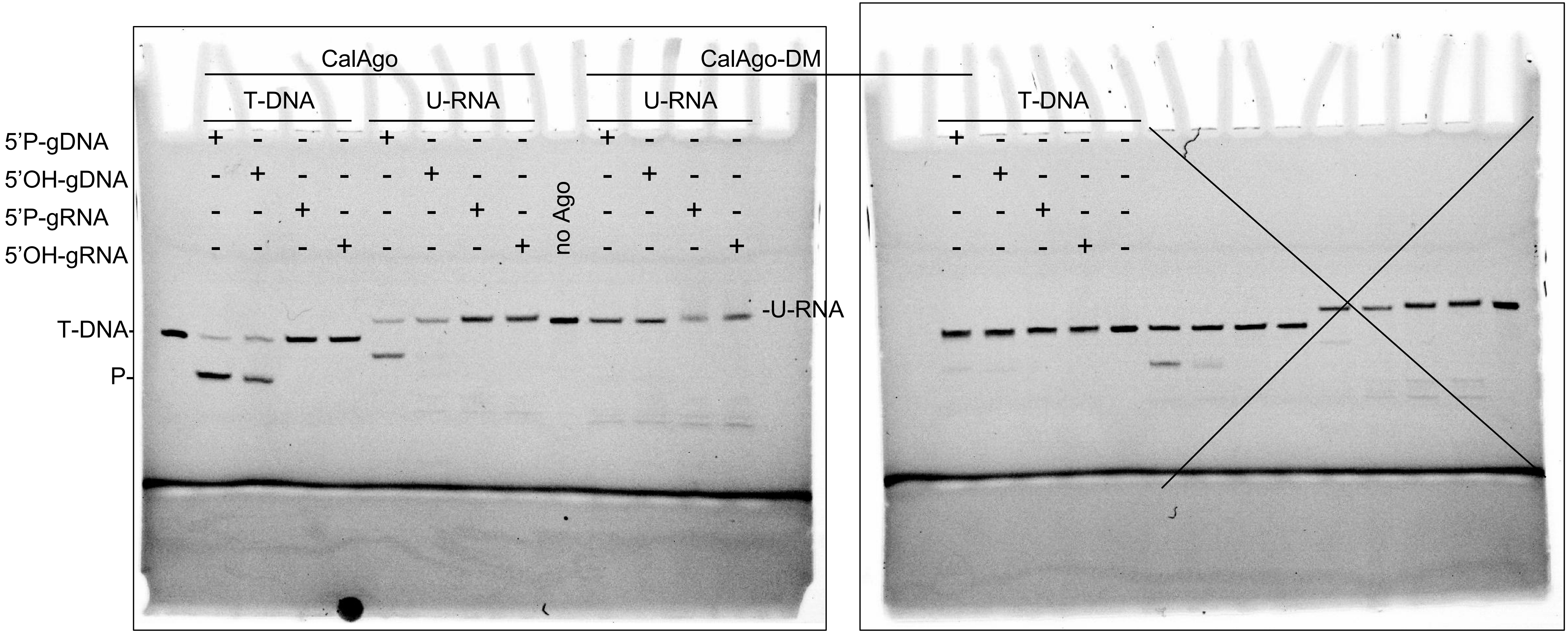

CalAgo
CalAgo-DM
T-DNA
U-RNA
U-RNA
T-DNA
5'P-gDNA
5'OH-gDNA
5'P-gRNA
5'OH-gRNA
no Ago
+ - - - + - - - + - - - + - - - -
- + - - - + - - - + - - - + - - -
- - + - - - + - - - + - - - + - -
- - - + - - - + - - - + - - - + -
-U-RNA
T-DNA-
P-

Figure 1C

Figure 2A

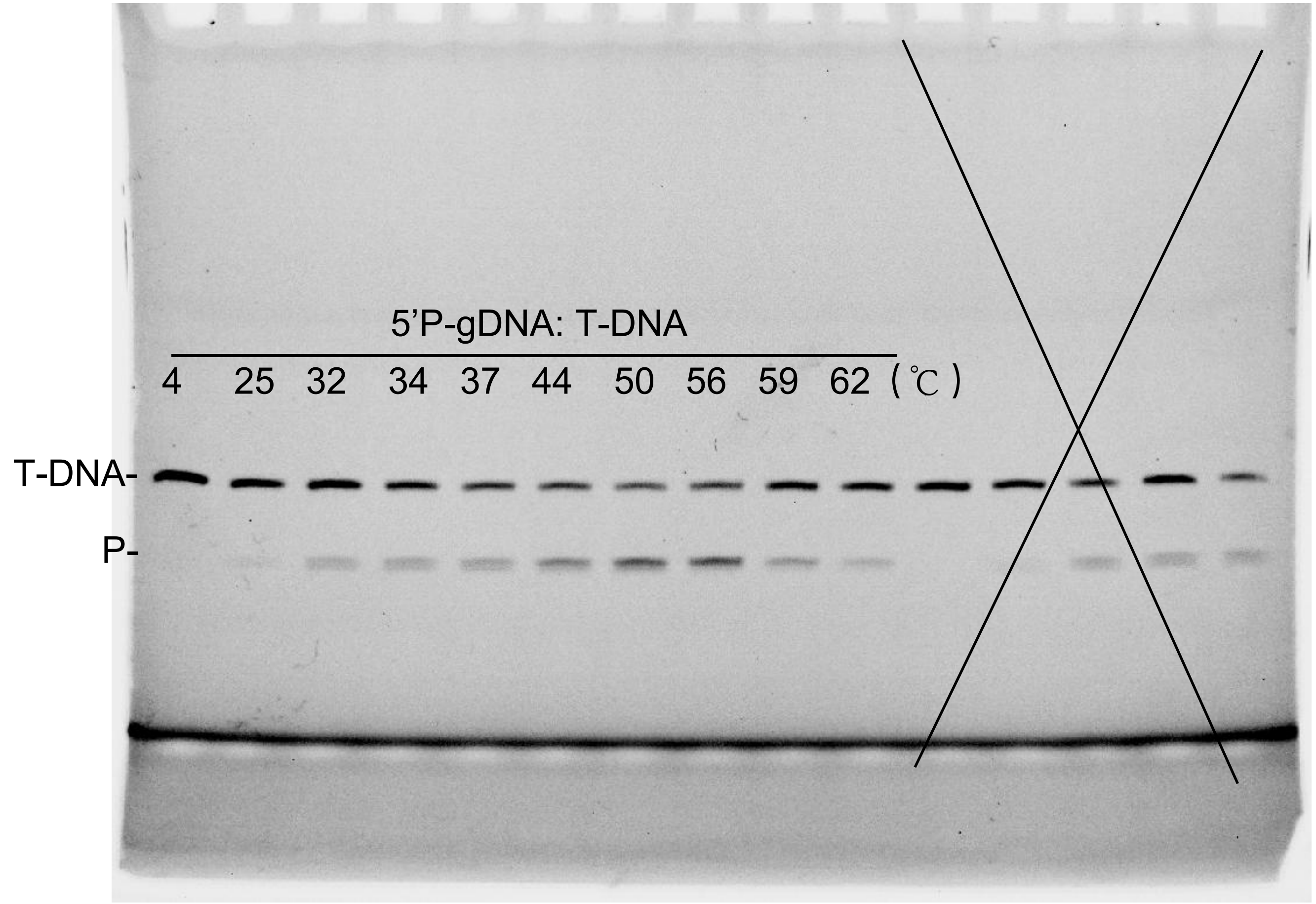

Figure 2B

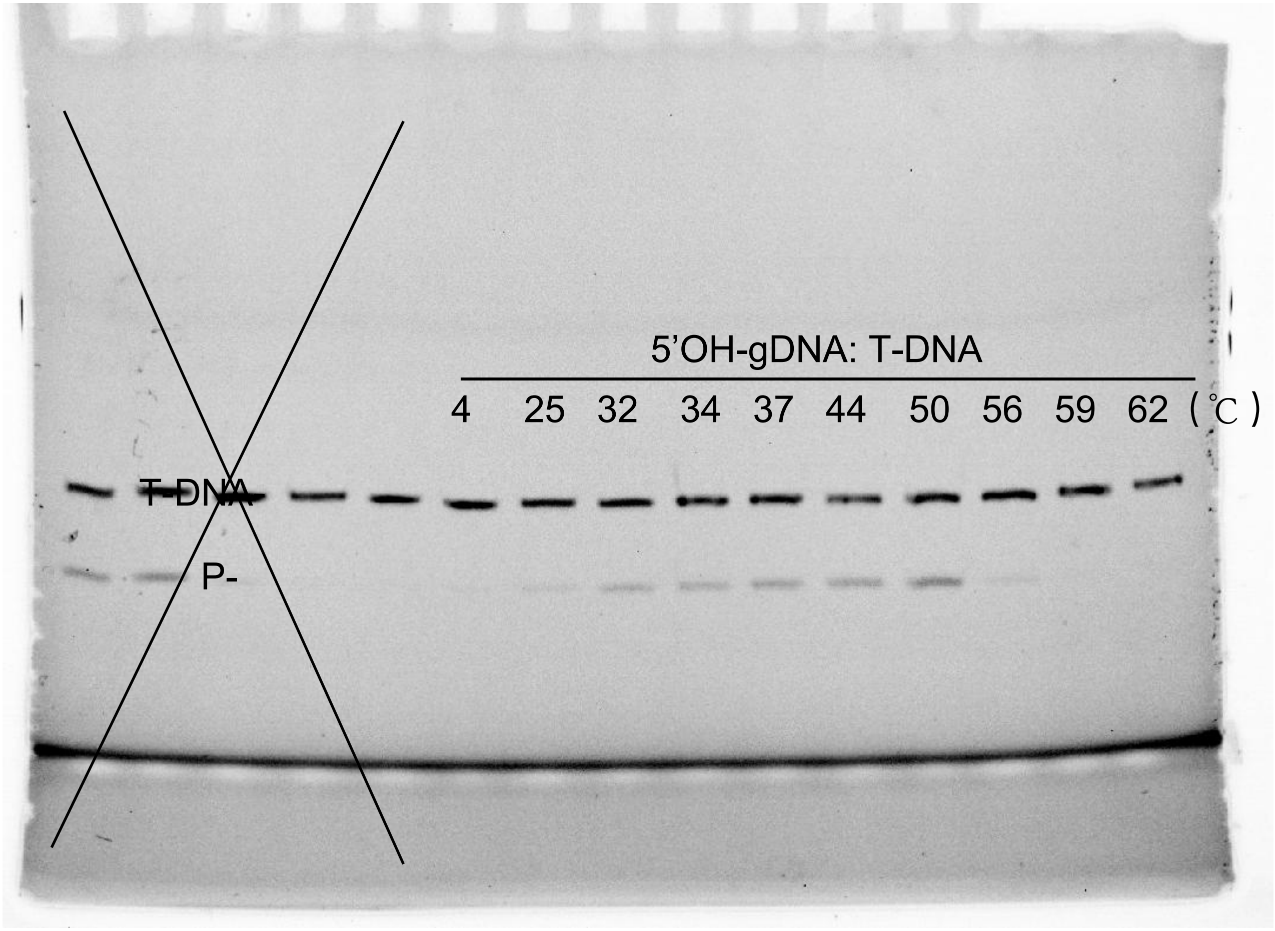

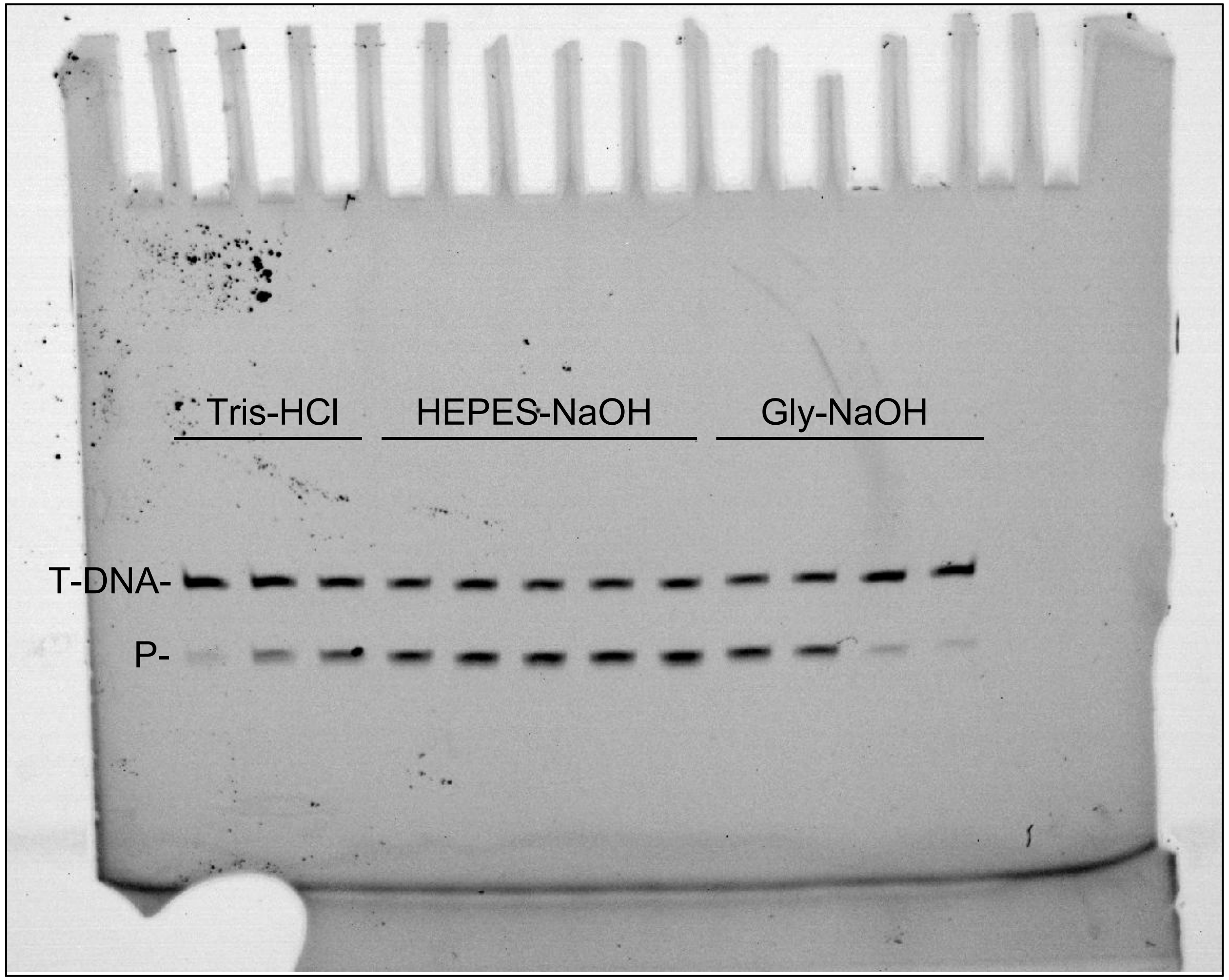

Figure 2C

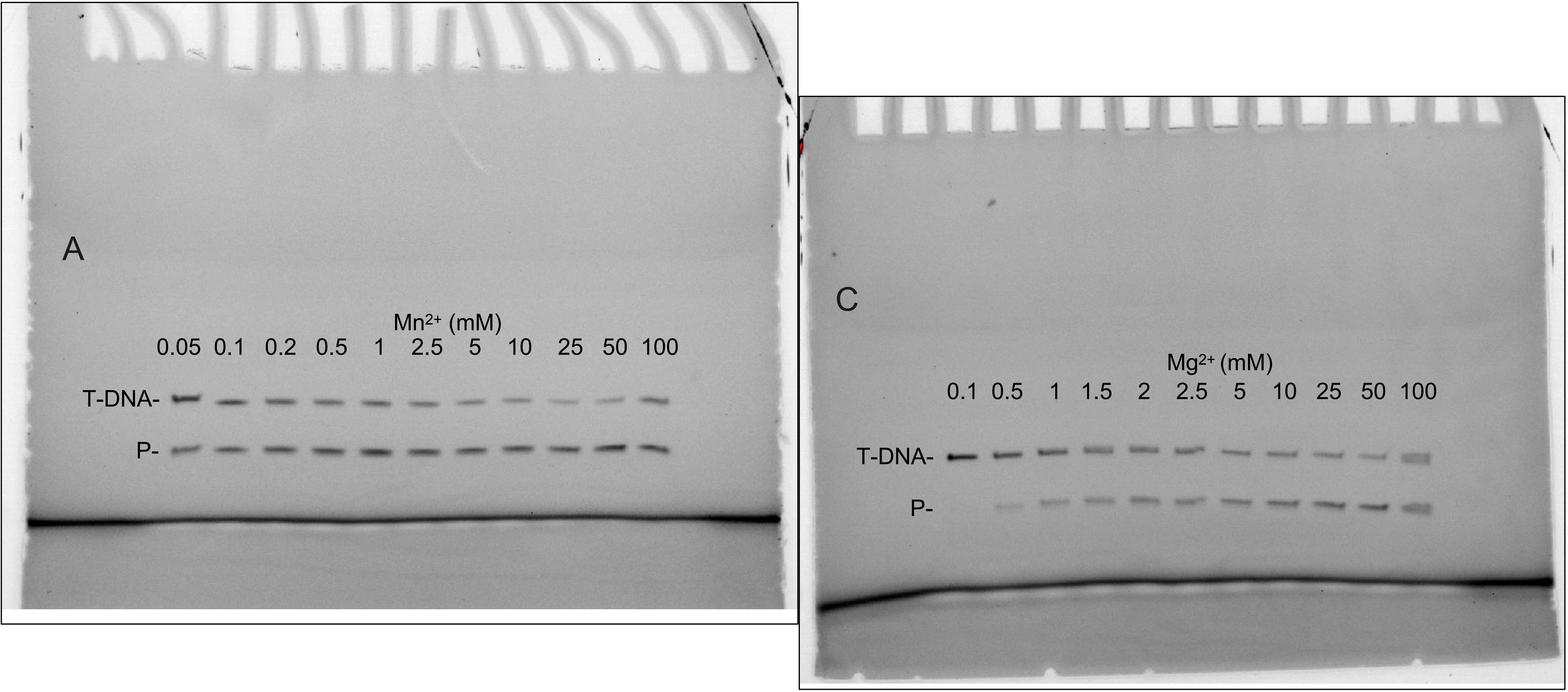
A
Mn2+ (mM)
0.05 0.1 0.2 0.5 1 2.5 5 10 25 50 100
T-DNA-
P-
C
Mg2+ (mM)
0.1 0.5 1 1.5 2 2.5 5 10 25 50 100
T-DNA-
P-

Figure 3A,C

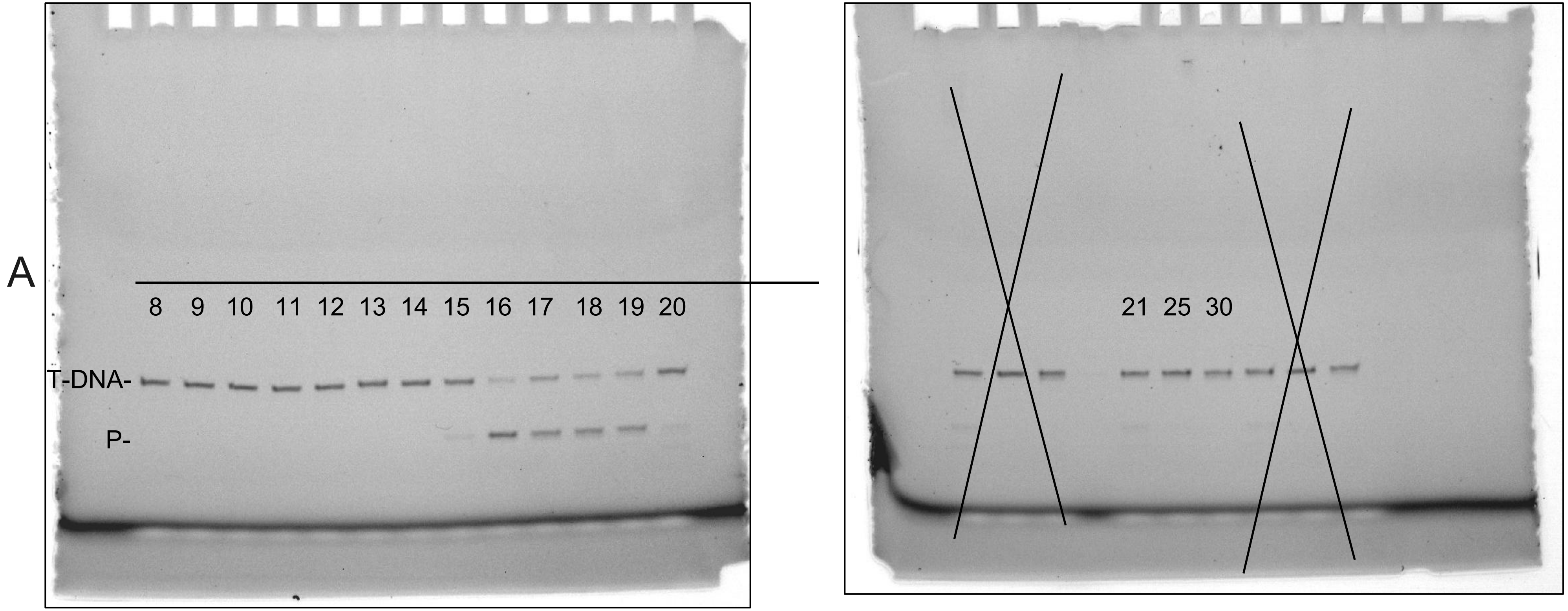

Figure 4A

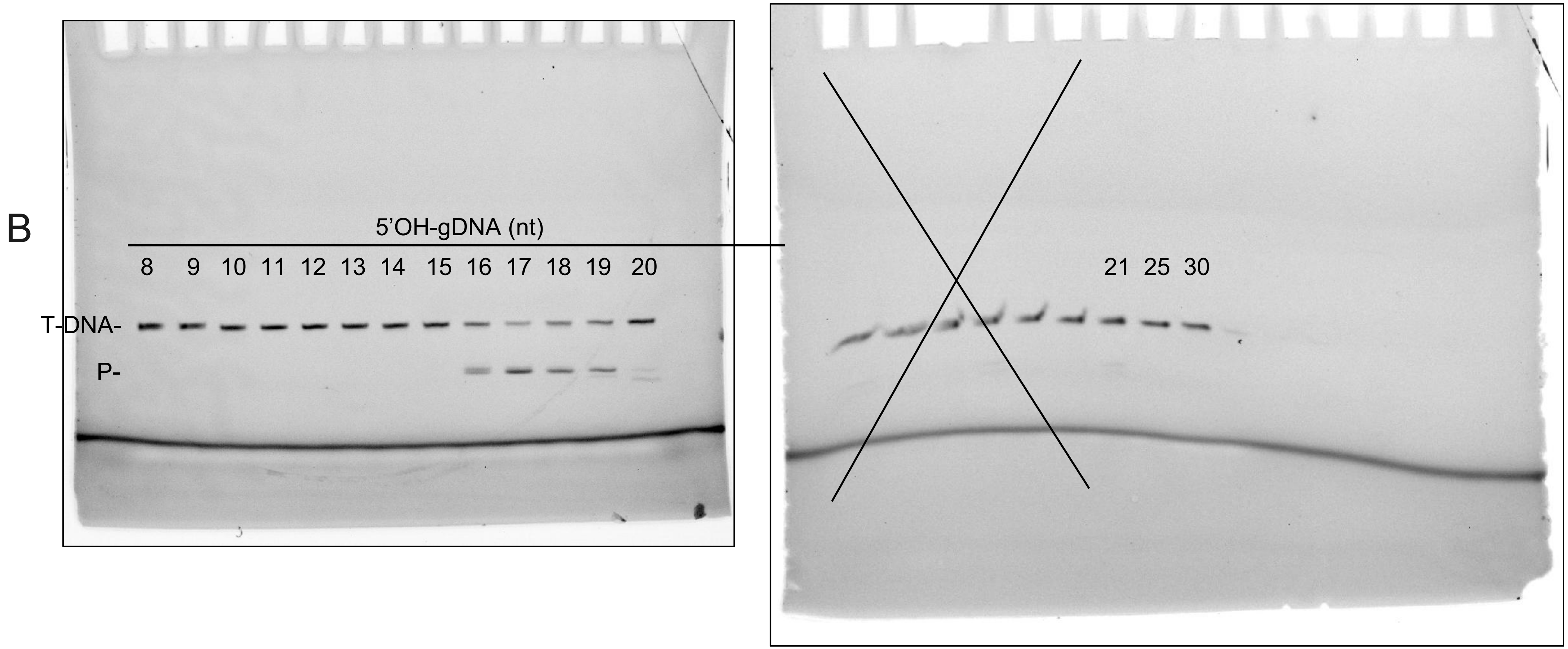

Figure 4B

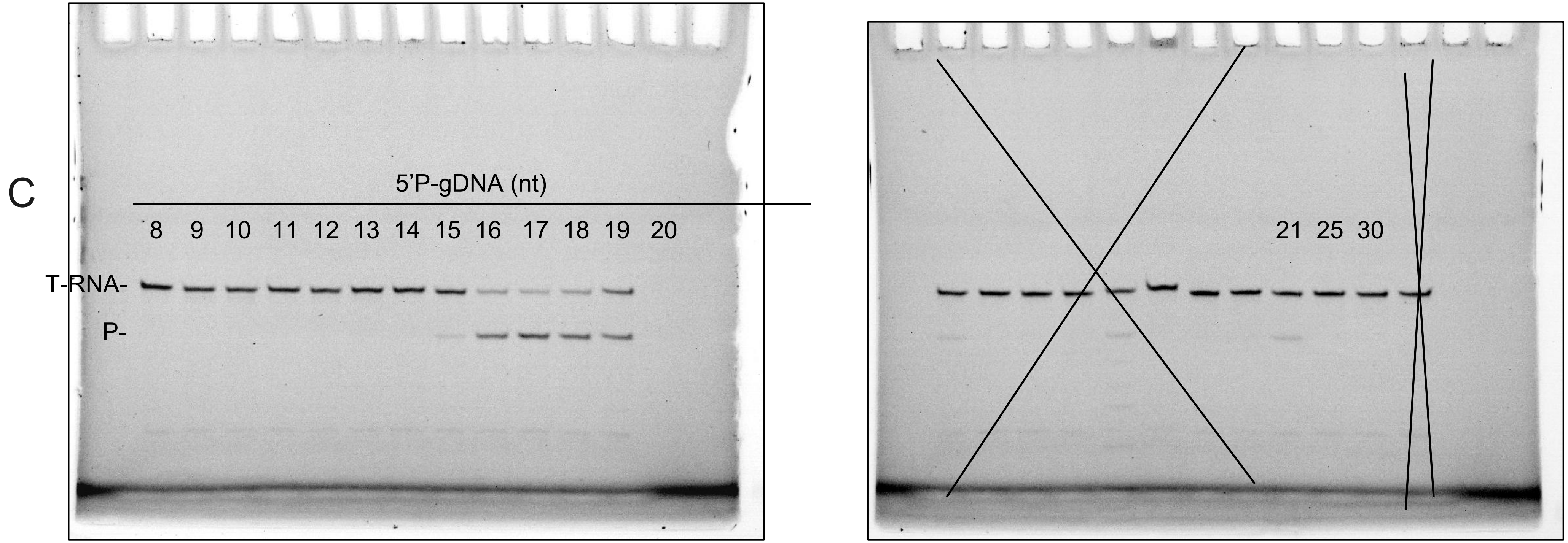

Figure 4C

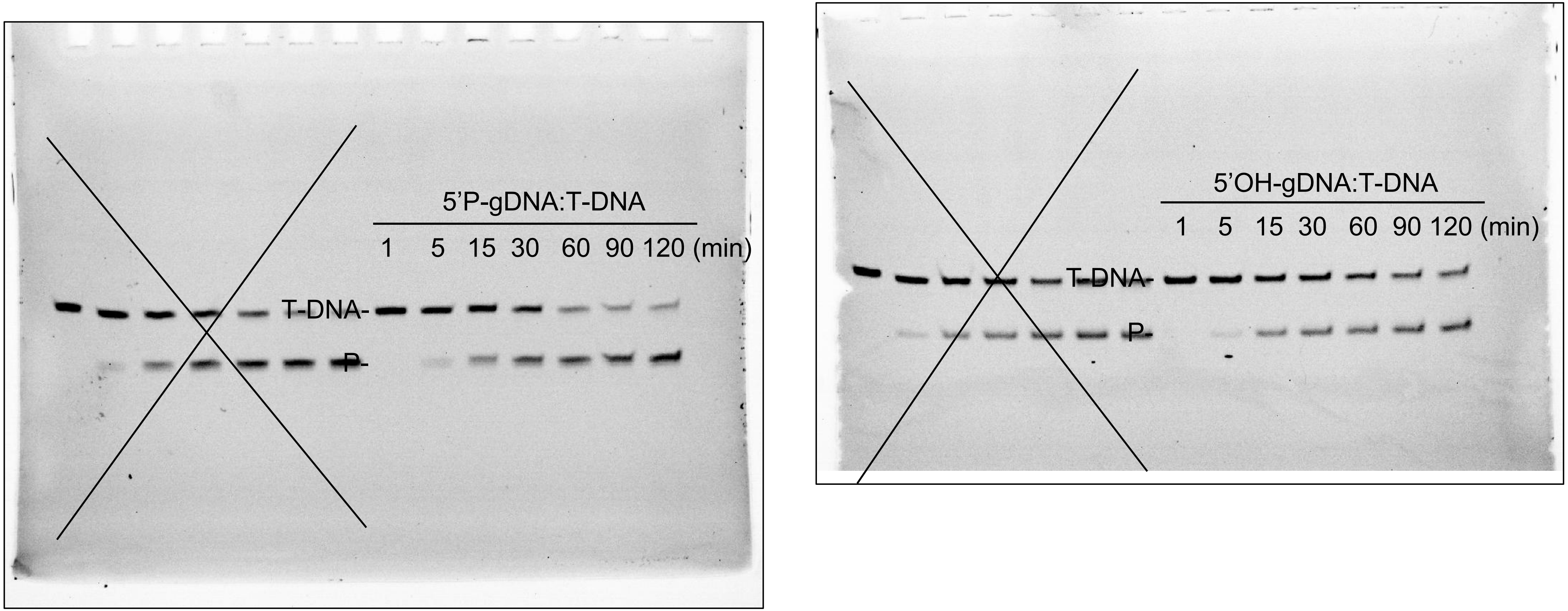
5'P-gDNA:T-DNA
1 5 15 30 60 90 120 (min)
T-DNA-
P-
5'OH-gDNA:T-DNA
1 5 15 30 60 90 120 (min)
T-DNA-
P-

Figure 5A

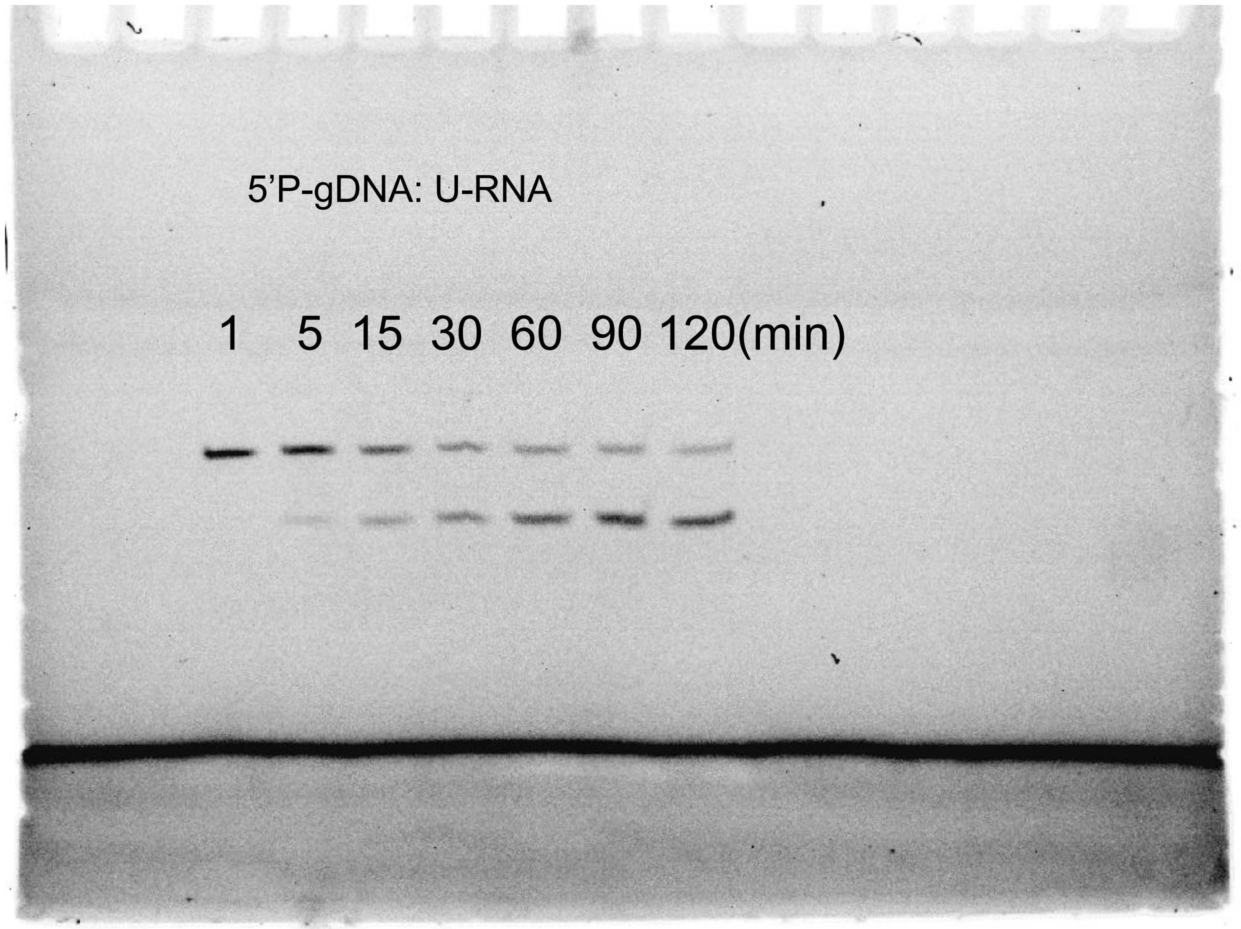

Figure 5A

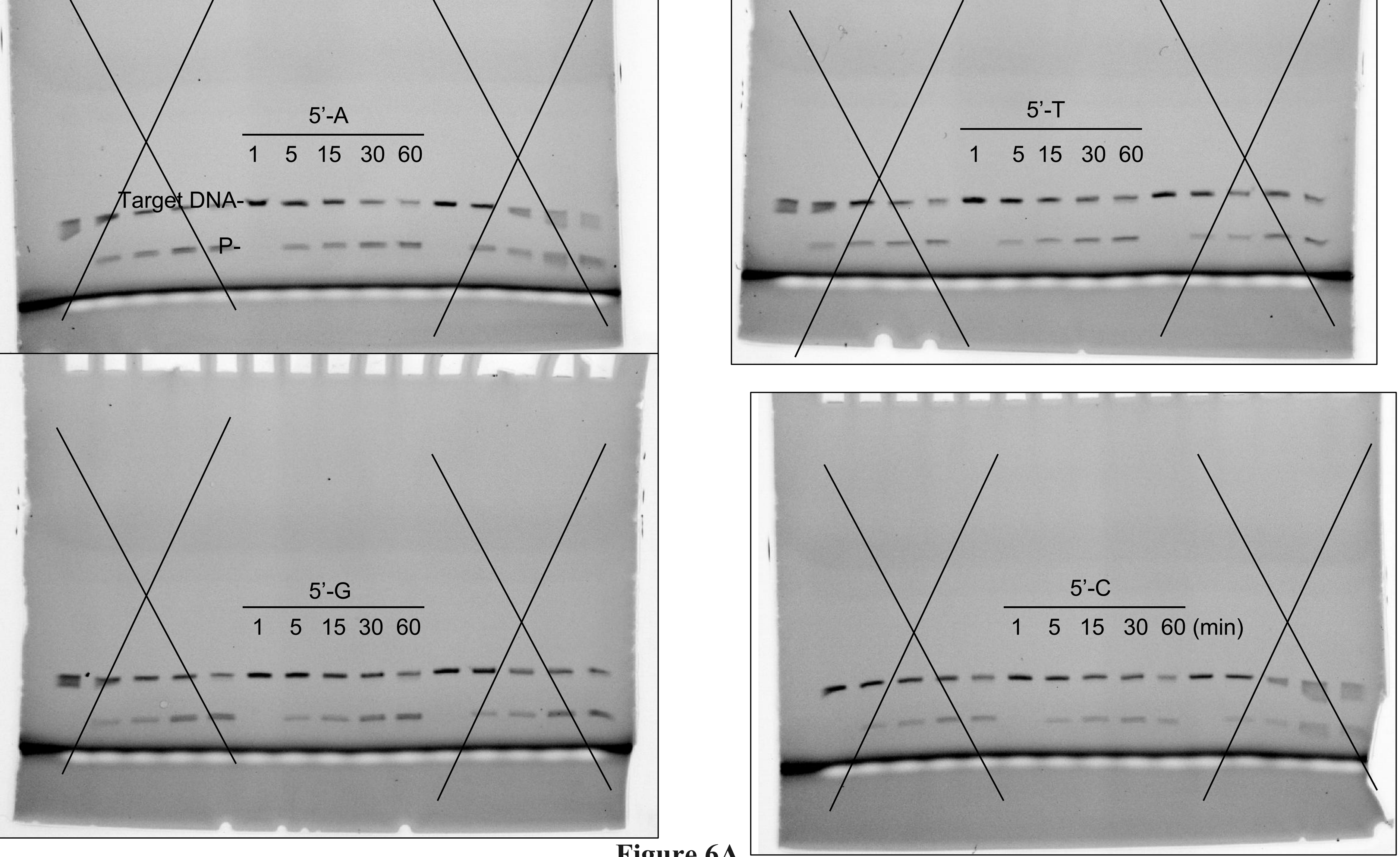
5’-A
1 5 15 30 60
Target DNA-
P-
5’-T
1 5 15 30 60
5’-G
1 5 15 30 60
5’-C
1 5 15 30 60 (min)

Figure 6A

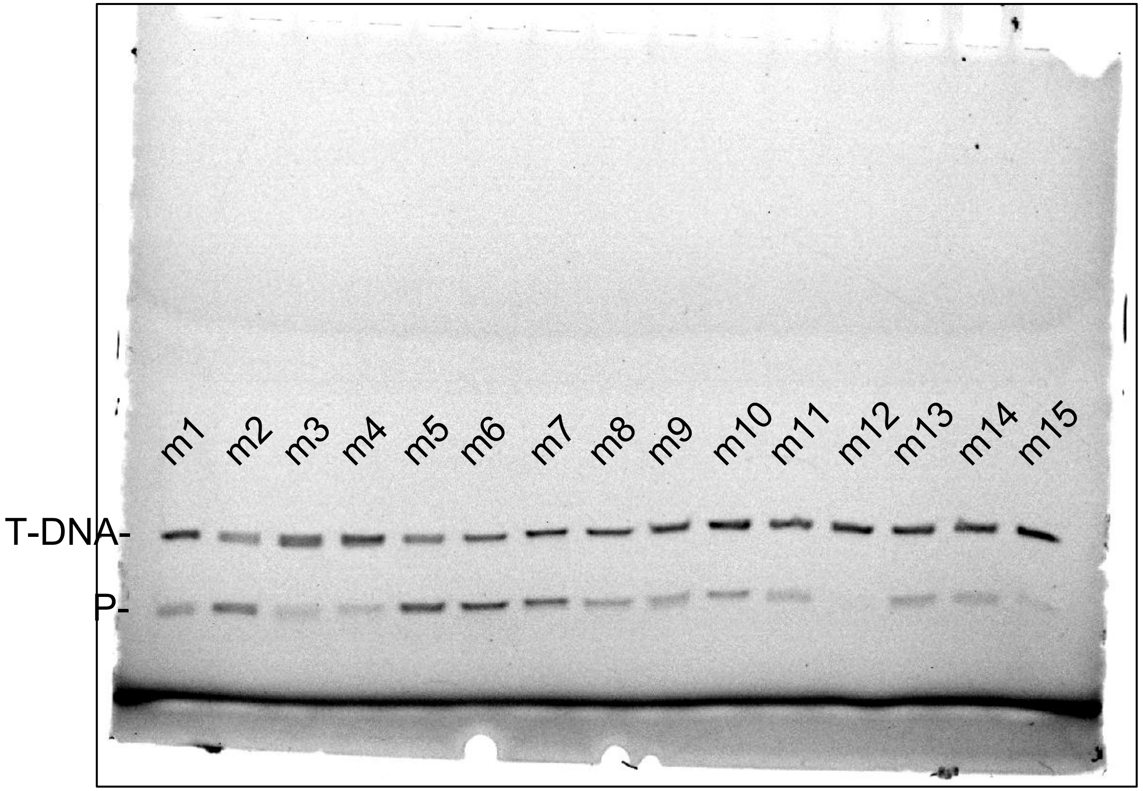

m1
m2
m3
m4
m5
m6
m7
m8
m9
m10
m11
m12
m13
m14
m15
T-DNA-
P-

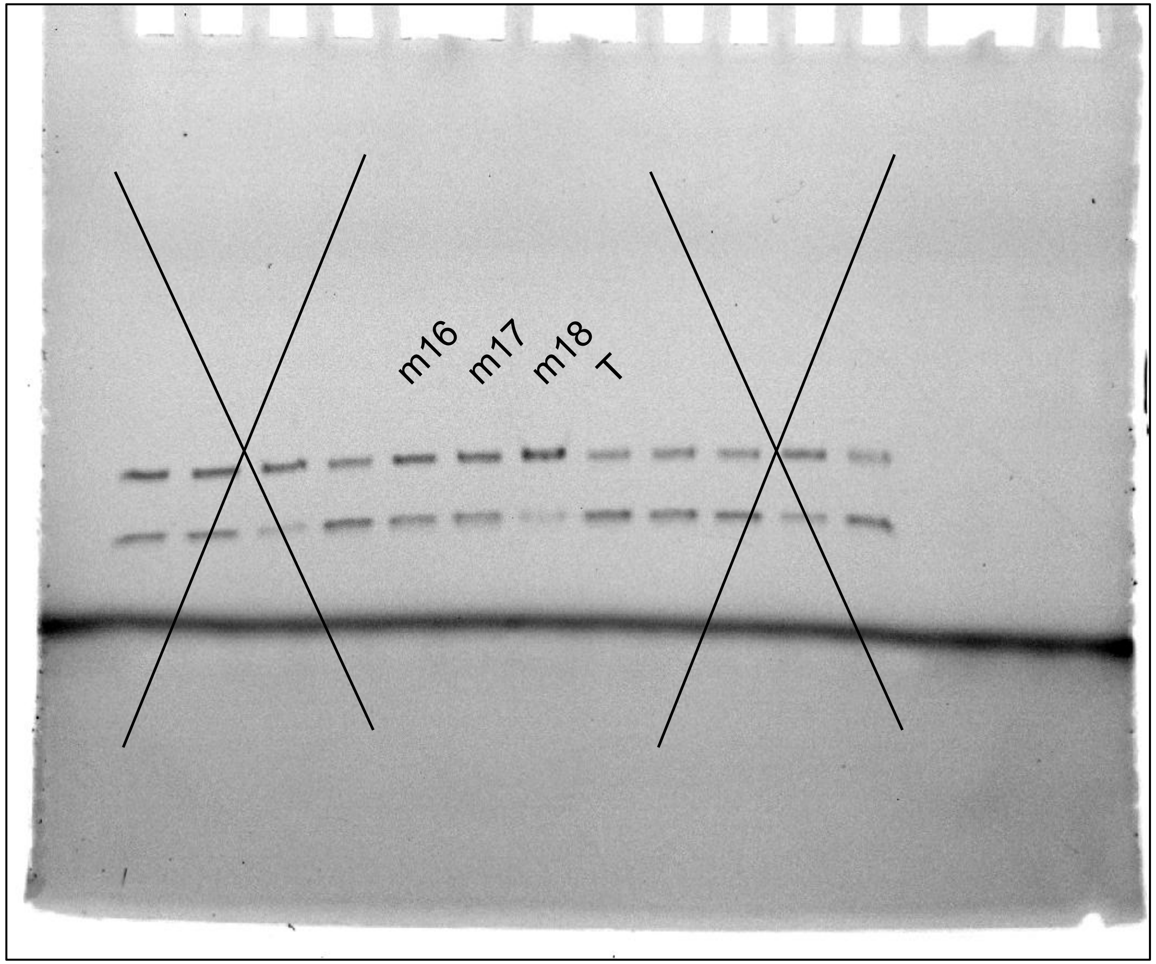

m16
m17
m18
T

Figure 7B

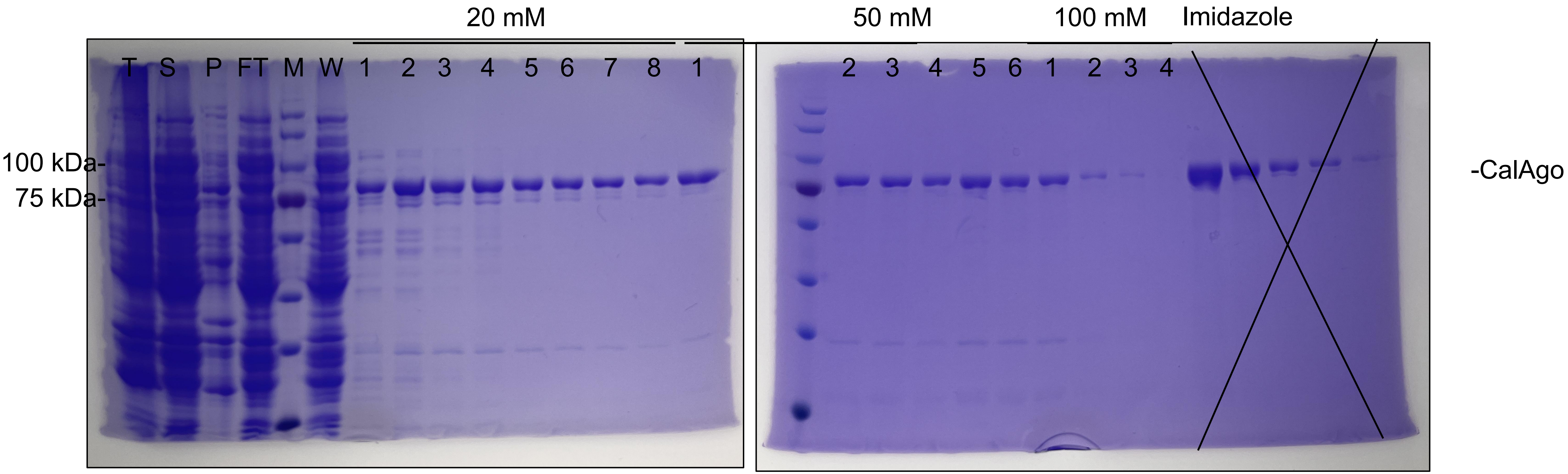
20 mM
50 mM
100 mM
Imidazole
T S P FT M W 1 2 3 4 5 6 7 8 1
2 3 4 5 6 1 2 3 4
100 kDa-
75 kDa-
-CalAgo

Figure S2A

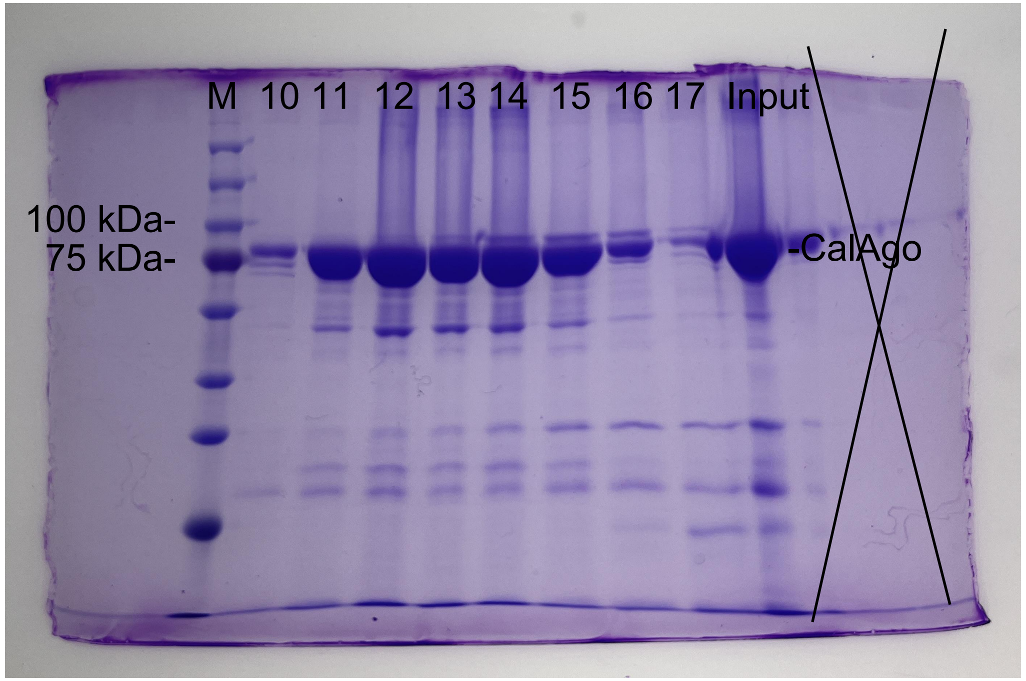

Figure S2B

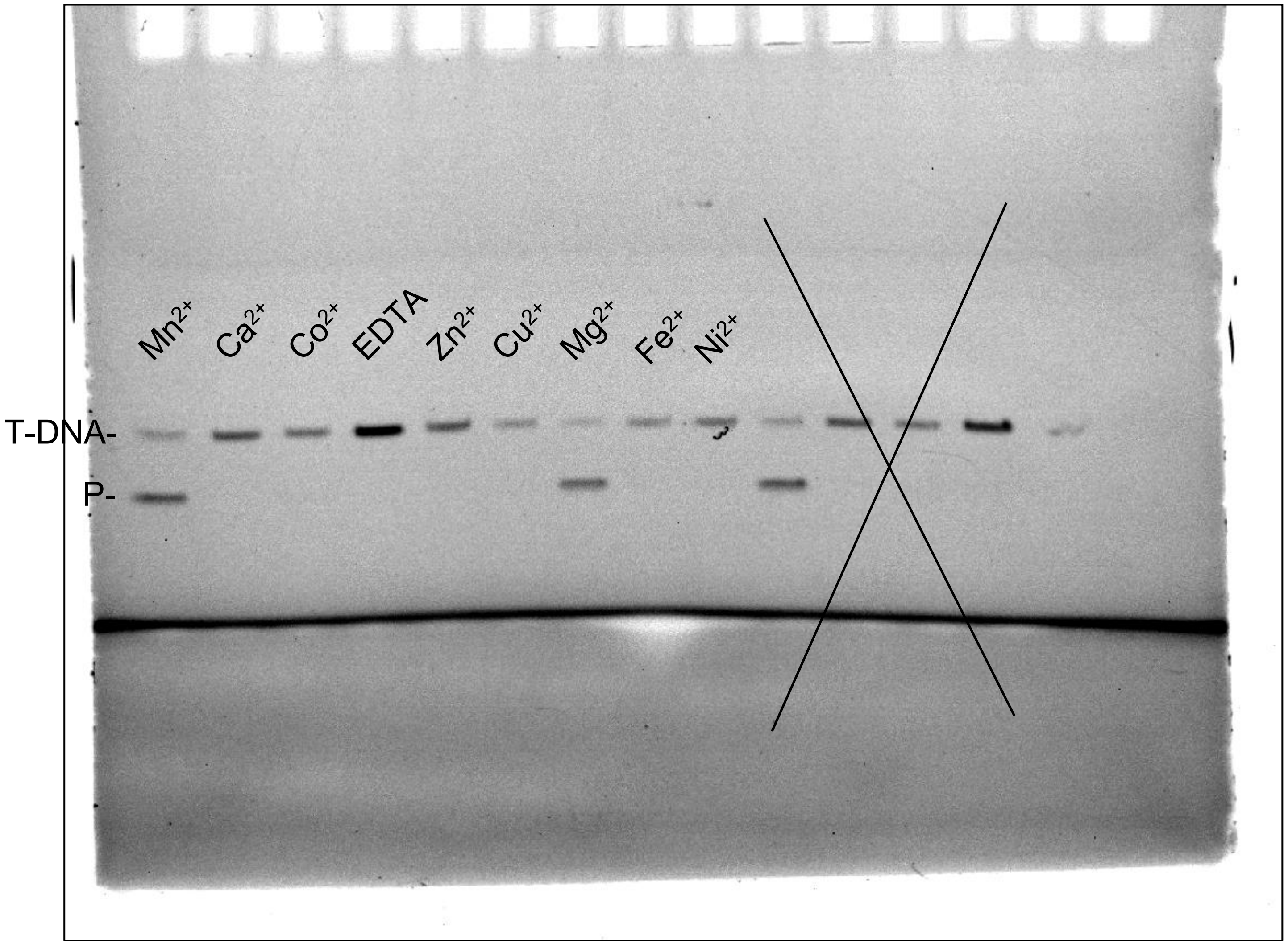

Figure S3

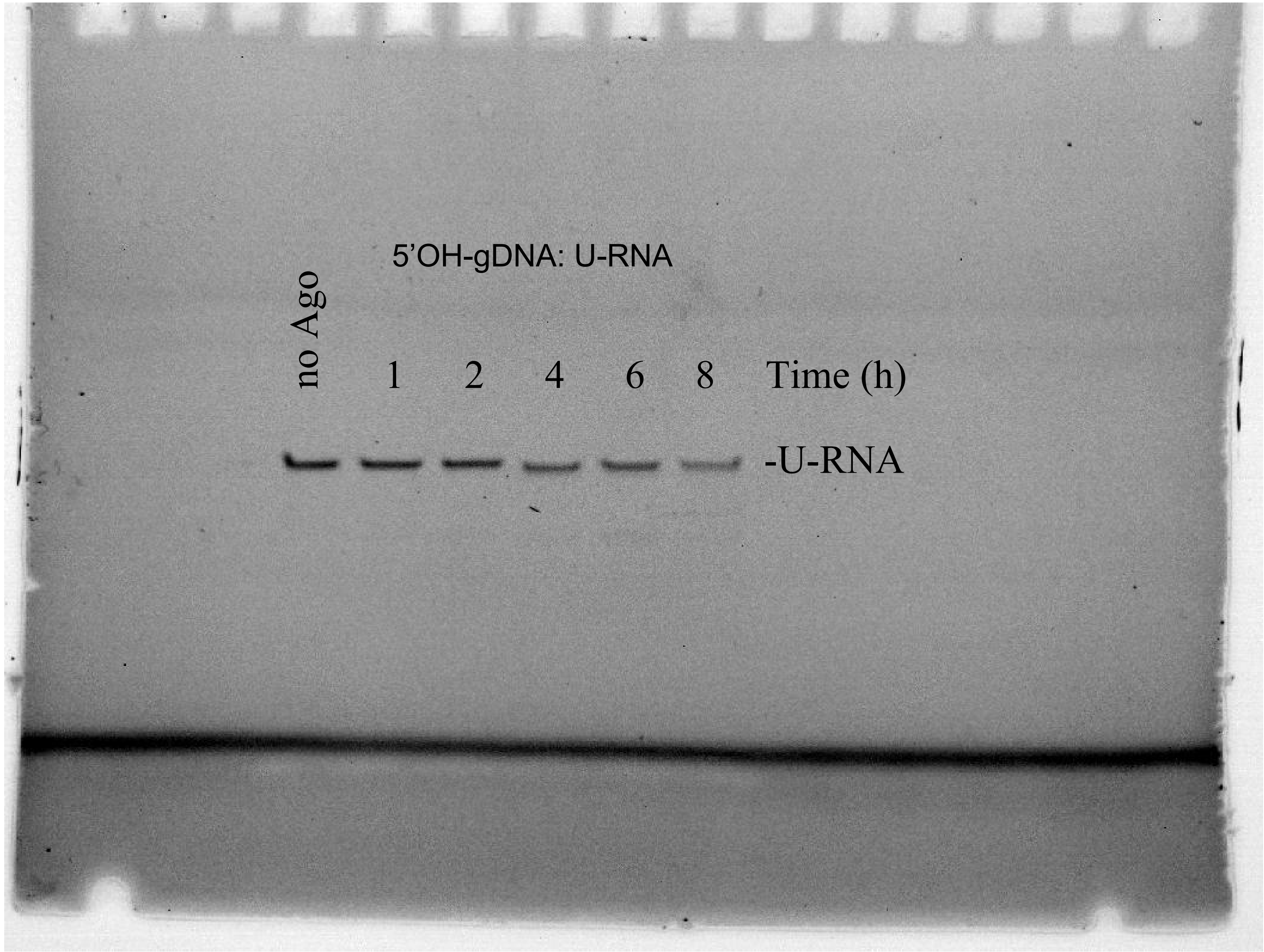

Figure S4

A

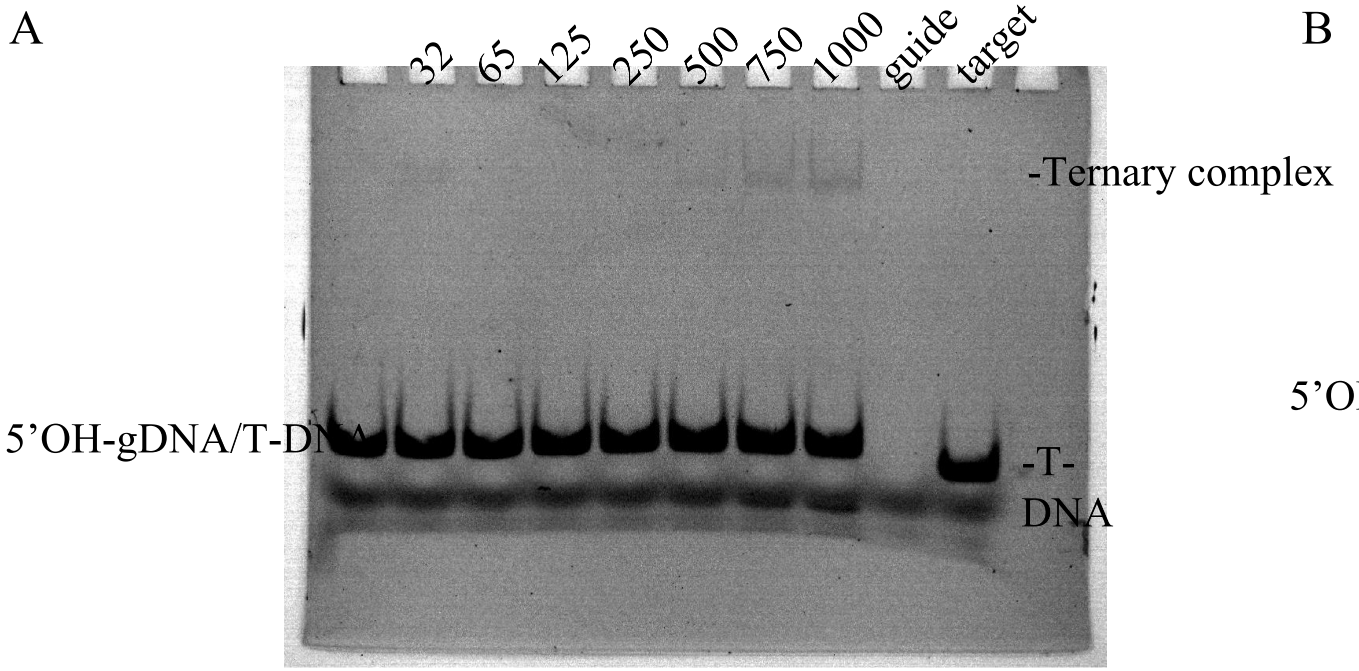
32
65
125
250
500
750
1000
guide
target
-Ternary complex
5’OH-gDNA/T-DNA
-T-
DNA

B

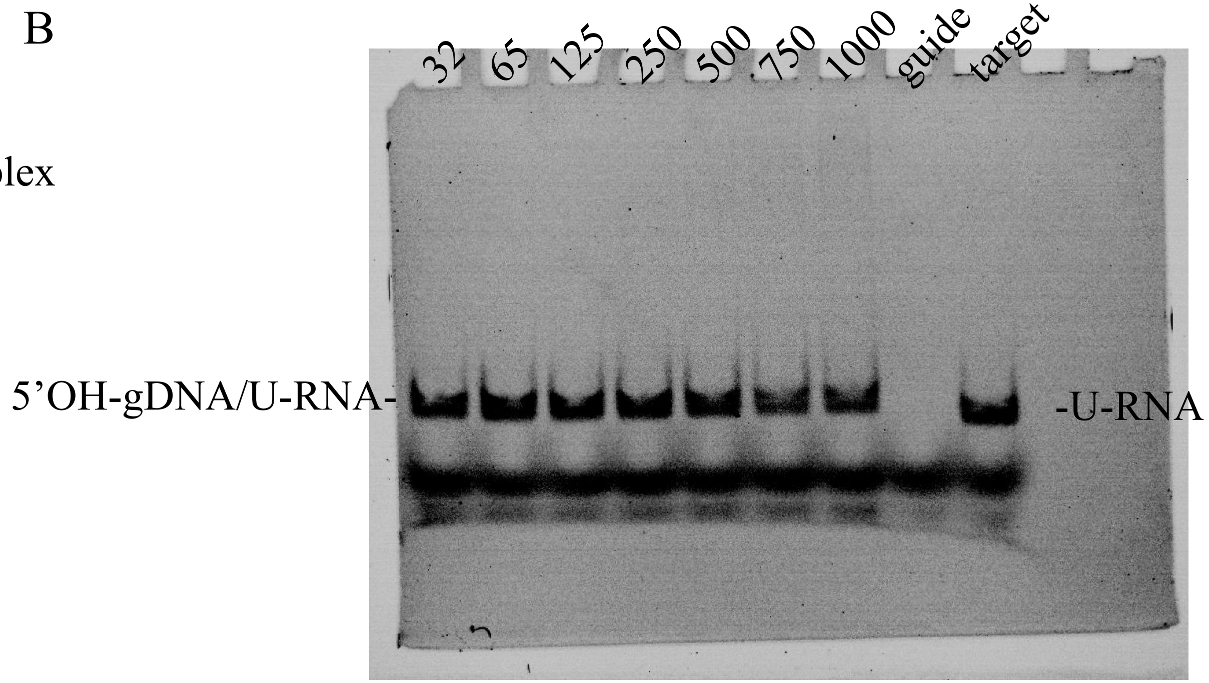
32
65
125
250
500
750
1000
guide
target
5’OH-gDNA/U-RNA-
-U-RNA

Figure S6
